# Surface Charge Transfer Enhanced Cobalt‐Phthalocyanine Crystals for Efficient CO_2_‐to‐CO Electroreduction with Large Current Density Exceeding 1000 mA cm^−2^

**DOI:** 10.1002/advs.202501459

**Published:** 2025-04-04

**Authors:** Tengyi Liu, Di Zhang, Yutaro Hirai, Koju Ito, Kosuke Ishibashi, Naoto Todoroki, Yasutaka Matsuo, Junya Yoshida, Shimpei Ono, Hao Li, Hiroshi Yabu

**Affiliations:** ^1^ Advanced Institute for Materials Research (WPI‐AIMR) Tohoku University Sendai 980‐8577 Japan; ^2^ AZUL Energy Inc. Sendai 980‐0811 Japan; ^3^ Graduate School of Environmental Studies Tohoku University Sendai 980‐8579 Japan; ^4^ Research Institute for Electronic Research (RIES) Hokkaido University Sapporo 001‐0020 Japan; ^5^ International Center for Synchrotron Radiation Innovation Smart Tohoku University Sendai 980‐8579 Japan

**Keywords:** cobalt phthalocyanine, electrochemical CO_2_ reduction, spray‐growth method, surface charge transfer

## Abstract

Phthalocyanines (Pcs) have garnered significant attention as promising catalysts for electrochemical CO_2_ reduction (ECR); however, traditional methods for preparing carbon‐supported Pcs are often complex and time‐consuming, limiting their industrial applicability. Herein, a rapid spray‐growth method is introduced that directly deposits CoPc crystals onto carbon paper (CP) in just 15 min. The resulting CoPc/CP electrode maintains > 90% CO selectivity across a broad ECR window (−0.57 to −1.32 V vs RHE), achieves a record‐breaking CO current density of −1034 mA cm^−2^, an ultrahigh mass activity of 5180 A g^−1^, and demonstrates excellent long‐term stability (145 h @ −150 mA cm^−2^), surpassing all reported Pc‐based catalysts. Comprehensive characterization attributes this high performance to its carbon‐supported‐crystalline structure and surface charge transfer (SCT). Density functional theory (DFT) calculations further reveal that even minimal SCT effectively optimizes the adsorption energies of key intermediates (^*^CO and ^*^COOH), thereby significantly enhancing intrinsic activity. Moreover, this spray‐grown electrode offers unique structural advantages, such as strong substrate adhesion and internal layers that replenish active sites—features absent in traditional carbon‐supported electrodes. It is believed that this facile spray‐growth method holds broad potential and enables the application of additional Pc‐based materials for industrial‐scale ECR.

## Graphical abstract

A spray‐growth method is developed to rapidly load cobalt‐phthalocyanine crystals onto carbon paper (CoPc/CP). The electrode maintains > 90% CO selectivity across potentials (−0.57–−1.32 V vs. RHE), achieving a record *J*_CO_ (−1034 mA cm^−^^2^), ultra‐high mass activity (5180 A g^−1^), and excellent stability (145 h@−150mA cm^−2^), surpassing all reported Pc‐based‐catalysts. DFT calculations confirm surface‐charge‐transfer and structural advantages significantly enhance the electrocatalytic performance of CoPc/CP electrodes.

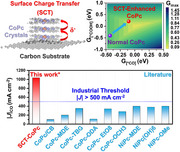

## 1 Introduction

As global energy demand and CO_2_ emissions rise, developing sustainable energy conversion and storage technologies is critical for mitigating climate change.^[^ ^1^, ^2^, ^3^ ^]^ Electrochemical CO_2_ reduction (ECR) technologies, which convert CO_2_ into valuable chemicals and fuels under mild conditions, offer a promising green approach toward carbon neutrality.^[^ ^2^, ^3^, ^4^ ^]^ Advancing ECR often requires electrocatalysts with high activity, stability, and adaptability for efficient, long‐term operation.^[^ ^2^, ^3^, ^4^, ^5^ ^]^ ECR involves multi‐electron transfers and multiple reaction steps, making it challenging for single‐component catalysts to achieve optimal binding energies at each stage.^[^ ^5^, ^6^ ^]^ Hybrid orbitals between metals and non‐metals, particularly metal‐nitrogen (M‐N_4_) coordination sites, have gained attention as effective strategies for enhancing electrocatalytic activity.^[^ ^7^, ^8^ ^]^ However, isolated M‐N_4_ sites often require specialized equipment and high‐temperature pyrolysis, and they may face stability issues under harsh conditions like high current densities (*J*) and strong electrolytes.^[^ ^7^, ^8^, ^9^ ^]^ Embedding M‐N_4_ sites in organic macrocyclic structures, such as metal‐porphyrins and metal‐phthalocyanines (M‐Pcs), can significantly enhance their stability and conductivity.^[^ ^9^, ^10^, ^11^ ^]^

M‐Pcs and their derivatives have gained more attention for their superior electrochemical properties compared to metal porphyrins, which is attributed to their higher content of pyridinic nitrogen.^[^ ^7^, ^9^, ^11^ ^]^ Moreover, their functionality or adaptability for various purposes can be tuned by modifying the central metals, attaching functional groups, or expanding the macrocyclic structure.^[^ ^7^, ^9^, ^11^ ^]^ This flexibility, diversity, and versatility give M‐Pcs materials significant potential in various reactions, including hydrogen evolution reaction (HER),^[^ ^12^, ^13^ ^]^ oxygen evolution reaction (OER),^[^ ^14^, ^15^ ^]^ oxygen reduction reaction (ORR),^[^ ^16^, ^17^ ^]^ and especially CO_2_ reduction reaction (CO_2_RR),^[^ ^4^, ^18^, ^19^, ^20^, ^21^ ^]^ where they excel in selectively producing valuable chemicals such as CO,^[^ ^18^, ^22^ ^]^ CH_4_,^[^ ^20^, ^21^ ^]^ CH_3_OH,^[^ ^4^, ^23^ ^]^ and C_2_H_4_,^[^ ^19^, ^24^ ^]^ etc. However, as semiconductor materials, phthalocyanines suffer from poor conductivity. Despite the traditional carbon‐supported methods can enhance their conductivity and dispersion, the preparation process is complex and time‐consuming, involving steps such as adsorption, filtration, and drying.^[^ ^18^, ^25^ ^]^ As a result, the achievable current densities and stability remain limited, restricting their industrial applications.^[^ ^18^, ^25^ ^]^ For example, reported maximum current densities just reach −400 mA cm^−2^, and the longest stability is 40 h.^[^ ^18^, ^26^ ^]^ Industrial applications, however, require higher reaction rates for longer‐term stability, with partial current densities exceeding 500 mA cm^−2^ and stability beyond 100 h.^[^ ^1^, ^27^ ^]^ Achieving these simultaneously on the electrode remains a significant challenge. Therefore, developing a rapid, efficient, and scalable fabrication method to produce high‐performance catalysts is crucial to advancing the industrial use of M‐Pc materials in ECR.

To overcome the limitations of traditional carbon‐supported methods, we developed an innovative spray‐growth technique incorporating ball‐milling and ultrafine spray‐coating. This approach directly loads M‐Pc crystals onto carbon paper (CP), reducing the preparation time from over 24 h to just 15 min. Systematic evaluation of various M‐Pc electrodes (Fe, Co, Ni, and Cu) for ECR revealed that CoPc exhibited significantly higher CO selectivity than other M‐Pc catalysts. Further studies demonstrate that this spray‐grown CoPc electrode achieves a record partial current density (*J*_CO_) of −1034 mA cm^−2^, ultra‐high mass activity of 5180 A g⁻¹, and exceptional long‐term stability of 145 h at −150 mA cm^−2^. These metrics surpass all previously reported Pc‐based catalysts for CO production, making it the first to exceed both the industrial threshold of 500 mA cm^−2^ and stability beyond 100 h. Moreover, high CO coverage is essential for effective C–C coupling; however, since CO is toxic, our hybrid, which serves as a promising CO_2_‐to‐CO producer in Pc‐tandem systems, offers significant advantages for high‐performance CO_2_‐CO‐C_2+_ conversion pathways.

Compared to conventional CoPc/KB materials, our spray‐grown CoPc crystals demonstrate a 7.6‐fold increase in intrinsic activity. Systematic characterizations indicate that this improvement stems from the carbon‐supported crystalline architecture and the spontaneous surface charge transfer (SCT) properties of the spray‐grown CoPc crystals. Moreover, density functional theory (DFT) calculations reveal that even minimal SCT induces negatively charged Co sites and substantially optimizes the binding energies of crucial intermediates (^*^COOH and ^*^CO), resulting in superior catalytic performance. In addition to high activity, our spray‐grown electrode exhibits distinct structural advantages over conventional carbon‐supported electrodes, including stronger substrate adhesion that ensures stability during extended electrolysis. Even if surface‐active sites detach, the catalyst's deeper layers effectively replenish them, maintaining overall performance. While enhancing M–Pc materials typically necessitate functional group grafting or macrocyclic expansions, however, we achieve such significant enhancement just with low‐cost CoPc using this novel spray‐growth method. This efficient technique could be broadly applicable, with the potential to enhance other M‐Pc materials for ECR.

## 2 Results and Discussion

### 2.1 Spray‐Growth Preparation of M‐Pc Electrode

**Figure 1:**
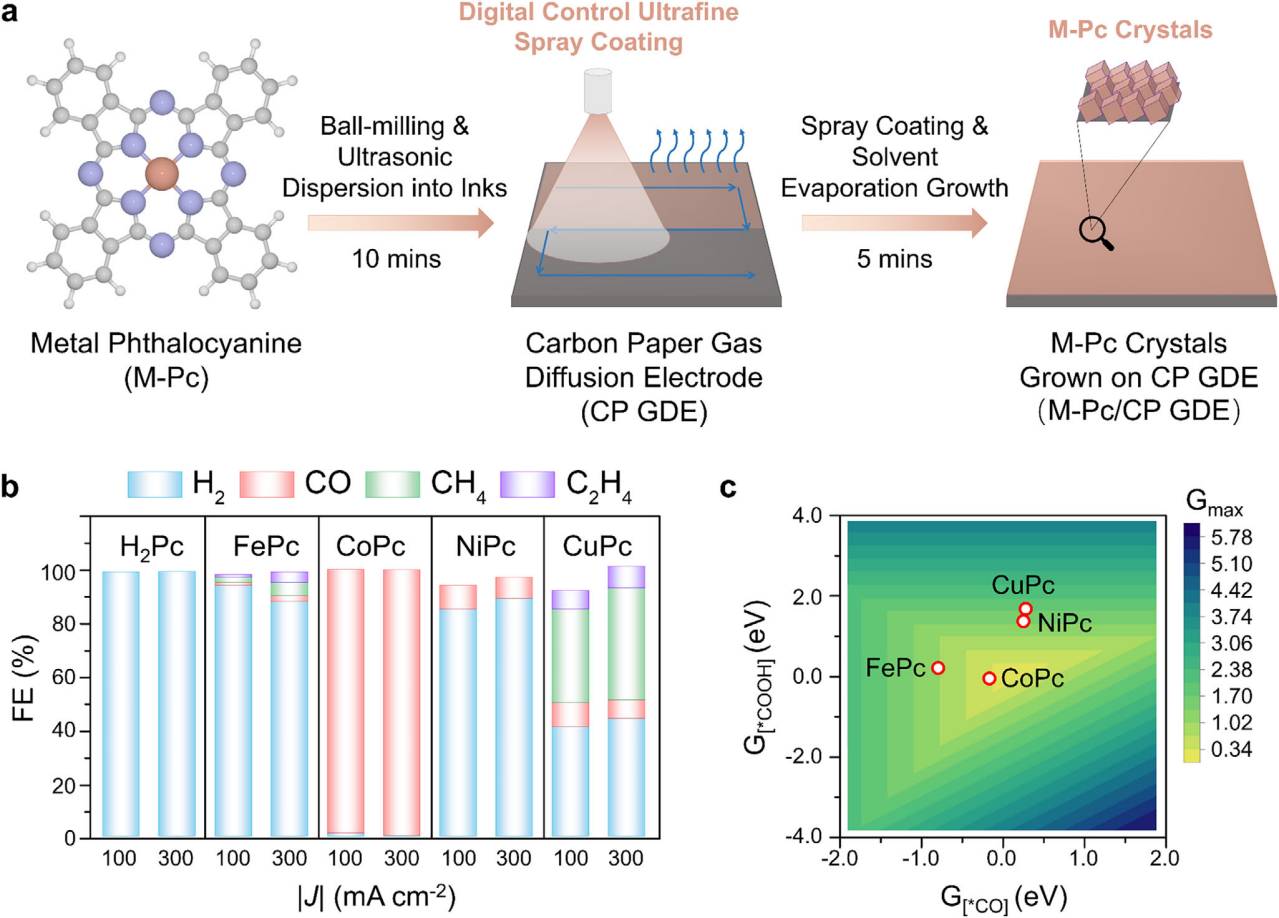
Rapid preparation of metal‐phthalocyanine (M‐Pc) electrodes and their electrochemical CO_2_ reduction (ECR) performance. a) Schematic of the 15‐min preparation for M‐Pc crystals on carbon paper gas diffusion electrodes (M‐Pc/CP GDEs). b) Comparison of selectivity at current densities of 100 and 300 mA cm^−2^ for ECR, using non‐metal phthalocyanine (H_2_Pc) and various M‐Pcs (FePc, CoPc, NiPc, and CuPc) GDEs. c) Volcano model with adsorption‐free energies (G_[*COOH]_ and G_[*CO]_) for various M‐Pc/carbon structures, comparing their theoretical activity in CO_2_‐to‐CO electroreduction.

Traditional carbon‐supported strategies for preparing M‐Pc electrodes typically involve adsorption, filtration, and drying steps. This process is complex, time‐consuming (>24 h), and requires careful handling to prevent contamination from organic solvents or carbon powder. To overcome these limitations, we developed a novel spray‐growth method. First, the M‐Pc powder undergoes ball milling with ZrO_2_ balls, which physically pulverize and disperse the catalyst; next, it is subjected to ultrasonic dispersion to create a uniform M‐Pc ink. The resulting ink is then directly spray‐coated onto carbon paper. This facile approach produces the M‐Pc electrode in just 15 min, significantly reducing time and cost. The preparation process is illustrated in **Figure** **1**a, and additional details, such as solvent types and ratios, can be found in the Experimental Section of the Supporting Information.

Using this spray‐growth method, we prepared several M‐Pc electrodes, including Fe‐, Co‐, Ni‐, and Cu‐based Pcs (chemical structures are shown in Figure S1, Supporting Information), and evaluated their performance for ECR in 1 m KOH. Figure 1b presents their electrochemical products and the corresponding Faradaic efficiency (FE), showing that CoPc exhibits significantly higher CO selectivity than the other catalysts. Interestingly, the non‐metal phthalocyanine (H₂Pc) shows no activity for ECR, indicating that the metal atoms are the active centers for the reaction, with Co sites demonstrating high intrinsic activity for CO production. DFT calculations reveal that different M‐Pc materials exhibit varying adsorption binding energies for intermediates (^*^COOH and ^*^CO). When these values are mapped onto a volcano model (Figure 1c), CoPc is positioned closer to the peak activity, which is consistent with the experimental results and confirms its higher intrinsic activity for CO_2_‐to‐CO electroreduction.

### 2.2 Characterization and Analysis

**Figure 2:**
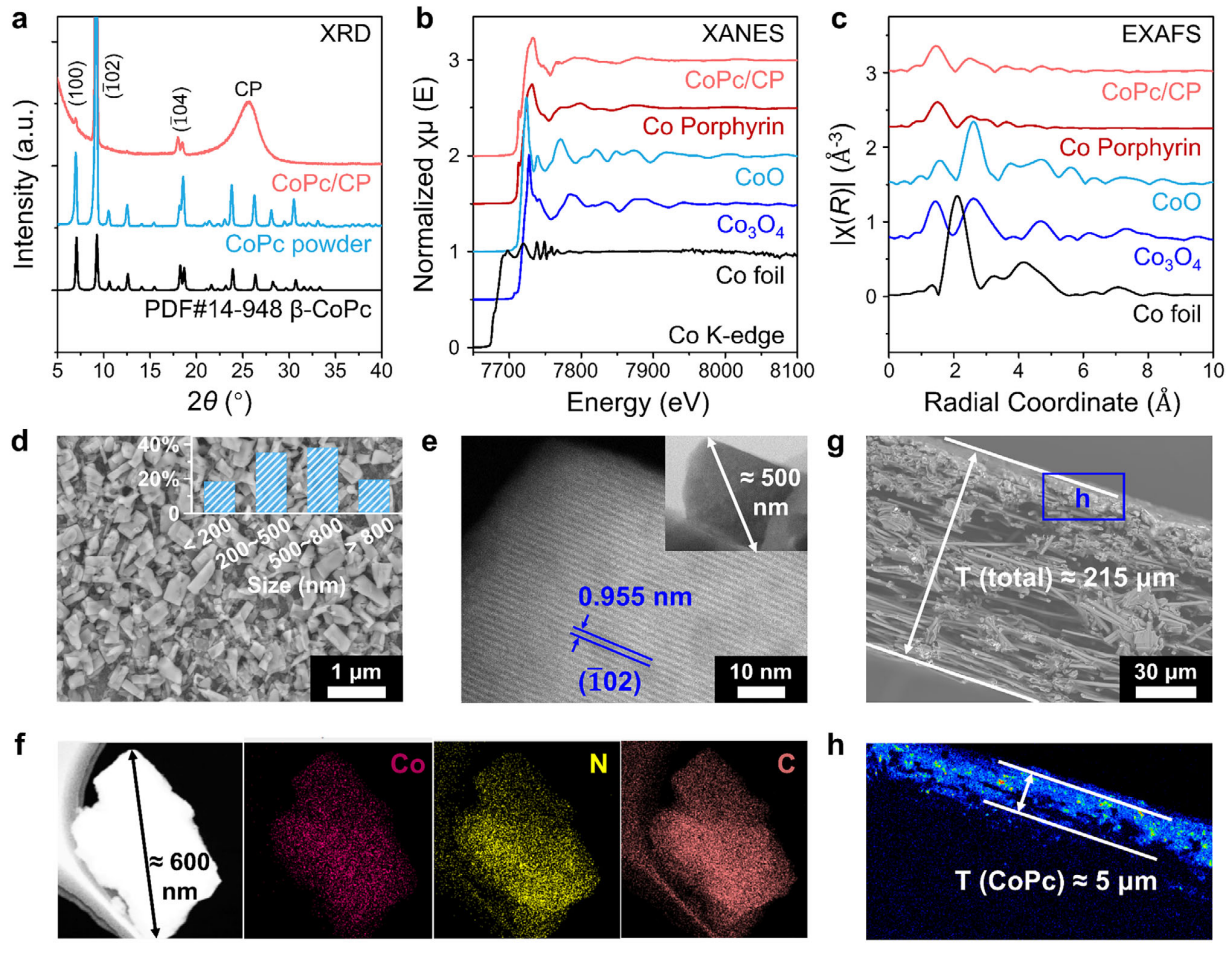
Morphological characterization and structural analysis of the spray‐grown CoPc electrode. a) XRD patterns of CoPc/CP and CoPc powder, compared with the standard beta‐CoPc card (PDF#No. 14‐948). b) XANES spectra at the Co K‐edge, and c) EXAFS spectra of CoPc/CP compared with reference samples (Co‐Porphyrin, CoO, Co_3_O_4_, and Co foil). d) HR‐SEM image of CoPc crystals with particle size distribution (inset), e) HR‐TEM image of a CoPc particle scratched from the CoPc/CP electrode, and f) elemental mapping images for Co, N, and C. g) Cross‐sectional SEM image, and h) EPMA image of CoPc/CP electrode from a cross‐sectional view.

X‐ray diffraction (XRD) analysis (**Figure** **2**a) indicates that the diffraction peaks of CoPc powder closely match the standard β‐CoPc pattern (PDF No. 14‐948). Notably, the spray‐grown CoPc crystals exhibit distinct diffraction peaks at 2θ values of 7.1°, 9.3°, and 18.2–18.7°, corresponding to the (100), (−102), and (−104) planes of β‐CoPc, respectively. The pronounced peak at 9.3° displays a very high relative intensity and sharp shape, indicating a preferred orientation along the (−102) plane. We infer that this phenomenon is closely related to the carbon substrate, in other words, the CoPc crystals align with the substrate during solvent evaporation. These findings provide solid evidence for a strong interaction between CoPc crystals and the substrate, which may enhance both stability and electrical conductivity.

To investigate the local electronic structure and chemical environment of Co centers, X‐ray absorption near‐edge structure (XANES) spectra of CoPc/CP and reference samples were recorded, as shown in Figure 2b. The peak shapes and energy absorption thresholds of CoPc crystals resemble those of Co porphyrin and are distinctly different from Co oxides, confirming the formation of Co‐N_4_ coordination structures. Extended X‐ray absorption fine structure (EXAFS) analysis further supports these findings, revealing similar coordination numbers and confirming that Co atoms are in a +2 oxidation state within the Pc molecules, forming stable Co‐N_4_ chelate structures (Figure 2c).

Figure 2d illustrates that scanning electron microscopy (SEM) images reveal the spray‐grown CoPc crystals have a granular morphology with an average particle size of 500–800 nm, covering ≈40% of the observed area. Scanning transmission electron microscopy (STEM) further shows clear lattice fringes in a CoPc particle detached from the CoPc/CP, indicating good crystallinity with a lattice spacing of 0.955 nm, consistent with the (‐102) plane of β‐CoPc, well corroborating the XRD results (Figure 2e). Elemental mapping confirms a uniform distribution of Co, N, and C, indicating the uniformity of the spray‐grown CoPc crystals (Figure 2f). Cross‐sectional SEM images indicate the total thickness of the electrode is ≈150 µm (Figure 2g), while electron probe micro‐analysis (EPMA) results reveal a uniform CoPc catalytic layer of about 5 µm thick on the CP substrate (Figure 2h).

Overall, the above characterizations confirm that the molecular structure of CoPc remains intact after ball‐milling and solvent evaporation, indicating the preservation of Co‐N_4_ sites. Moreover, the CoPc crystals exhibit a preferred orientation, growing along the substrate, which suggests strong adhesion and enhanced electron transfer capability. These results strongly indicate that the successful preparation of the spray‐grown CoPc electrode shows great potential as an efficient catalyst for ECR.

### 2.3 Electrocatalytic Performance of CO_2_ Reduction

To evaluate the ECR performance of these spray‐grown CoPc electrodes, the corresponding GDEs were pre‐treated and used as cathodes in a three‐electrode flow‐cell electrolyzer, with 1.0 m KOH as the electrolyte. The detailed ECR system and flow‐cell setup are shown in Scheme S1 (Supporting Information). Gas products were analyzed using gas chromatography with flame ionization detection (GC‐FID), and the calibration procedures for four standard gases (CO, CH_4_, C_2_H_4_, and H_2_), along with the corresponding fitting lines, are presented in the Experimental Section, Table S1, and Figures S2 and S3 (Supporting Information).

**Figure 3:**
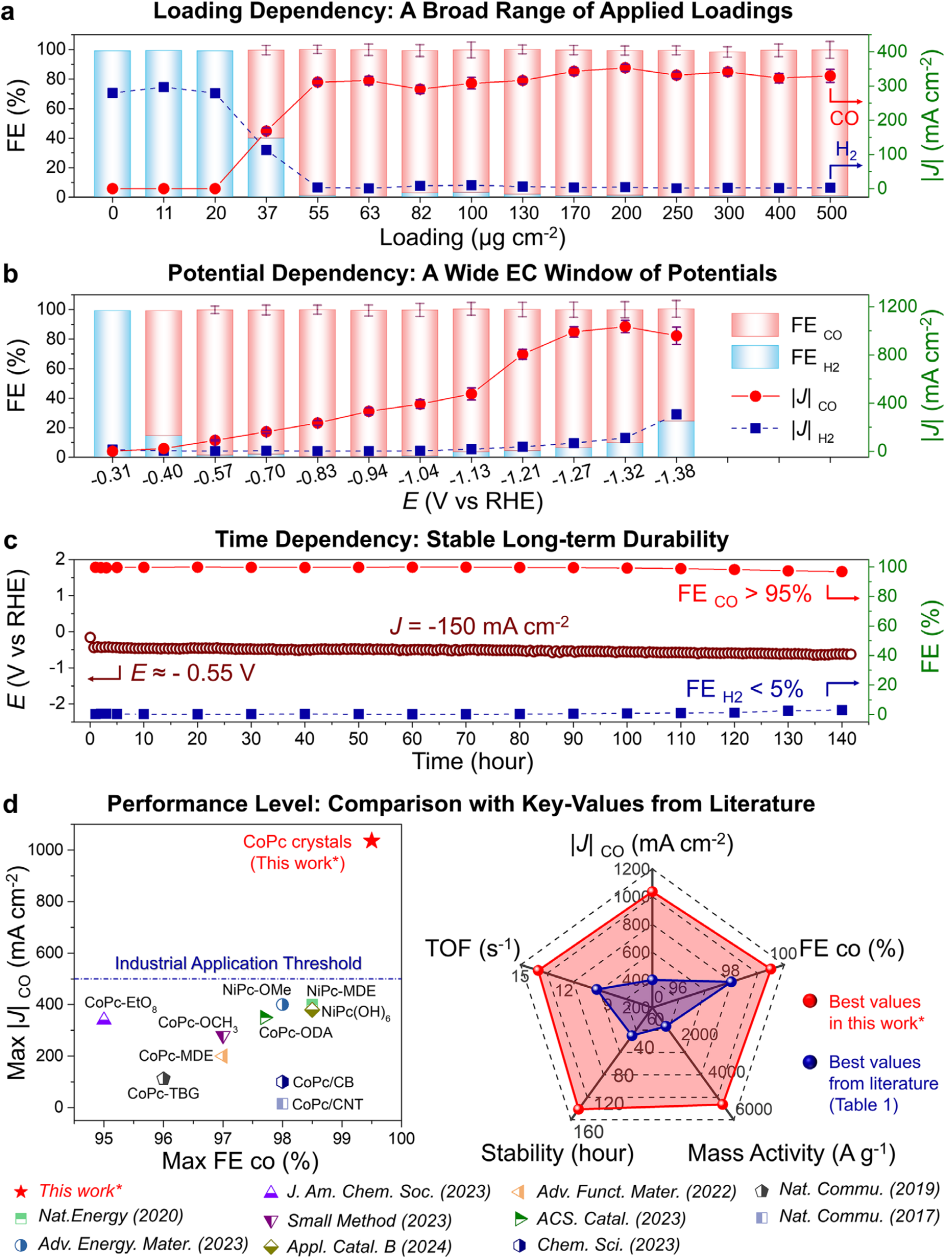
Electrochemical performance of various GDEs for ECR. a) Faradic efficiency (FE, left axis) and absolute current density (|*J*|, right axis) for CO (red) and H_2_ (blue) for electrodes at different loadings, at a potential (*E*) of −0.94V (vs RHE). b) FE (left axis) and |*J*| (right axis) as a function of *E* (vs. RHE) for electrodes at a loading of 200 µg cm^−2^. c) Long‐term stability of the CoPc/CP electrode at a constant *J* of −150 mA cm^−2^., showing variation in *E* (left axis) and FE (right axis). d) Comparison of maximum FE and |*J*| for CO in this work with literature values (left), and radar plots showing key metrics from this work (red) versus the literature (blue) (right).^[^ ^18^, ^25^, ^29^, ^30^, ^31^, ^32^, ^33^, ^34^, ^35^ ^]^

**Figure** **3**a shows the ECR results for CoPc crystals at various loadings. At lower CoPc loadings, hydrogen (H₂) is the predominant product due to the inherently low ECR activity of the CP substrate. As the CoPc loading increases, CO selectivity significantly improves, reaching a faradaic efficiency (FE) of over 98% for CO at a loading of 55 µg cm^−2^. Further increasing the loading enhances the total current density (*J*_total_), primarily due to the increased catalytic surface area and number of active sites. At an optimal loading of 200 µg cm^−2^, *J*_total_ reaches its maximum, and the CO current density (*J*_CO_) attains −350.3 mA cm^−2^. Interestingly, when the loading exceeds 200 µg cm^−2^, the *J*_CO_ values do not increase, likely due to diffusion limitations of CO_2_ in the electrolyte. At this point, the three‐phase boundary layer comprising the catalyst, electrolyte, and CO_2_ gas becomes saturated with accessible active sites. Further increases in loading may reduce conductivity, leading to a slight decline in *J*_CO_. Thus, 200 µg cm^−2^ is identified as the optimal loading for CoPc/CP catalysts.

To explore the relationship between potential (*E*) and ECR performance, CoPc/CP GDEs with a loading of 200 µg cm^−2^ were evaluated across various potentials (Figure S4, Supporting Information). According to the literature, the CO_2_ reduction to CO is a two‐electron transfer process with a theoretical *E* of −0.52 V (vs SHE), equivalent to −0.11 V (vs RHE) in 1 m KOH solution (pH 14) (Table S2, Supporting Information).^[^ ^28^, ^29^ ^]^ In practical applications, an additional overpotential is needed to overcome the reaction's energy barrier. The competing HER has a much lower theoretical *E* of 0 V (vs RHE), making it more favorable under similar conditions. Therefore, to effectively suppress HER and enhance CO selectivity, catalysts must exhibit high intrinsic activity specifically for ECR.

Figure 3b illustrates that at *E* = −0.31 V (vs RHE), H₂ is the primary product (see Table S3 and Figure S5, Supporting Information for more details). As the potential shifts to −0.40 V, CO emerges as the dominant product with an FE of 80%. At *E* = −0.57 V (vs RHE), FE_CO_ surpasses 98%, indicating that CO_2_RR is the prevailing reaction while HER is significantly suppressed. Even at a more negative potential of −1.04 V (vs RHE), FE_CO_ still remains above 98%, showcasing the excellent selectivity of these spray‐grown CoPc crystals for ECR and their effective suppression of HER across a broad potential range.

As the potential increases, *J*_CO_ rises sharply, peaking at −1036 mA cm^−2^ at *E* = −1.32 V (vs RHE), significantly surpassing all electrocatalysts reported to date. According to the equation *J*_partical_ = *J*_total_ × FE, the high *J*_CO_ can be attributed to both a high *J*_total_ and a high FE. The high *J*_total_ is influenced by the number of active sites or the thickness of the three‐phase interface, while the high FE is linked to the catalyst's intrinsic electrocatalytic activity. Therefore, a high *J*_CO_ of CoPc/CP reflects both their structural advantages and superior catalytic activity.

**Table** **1** summarizes the performance parameters of classic M‐Pc materials reported in recent years as CO_2_‐to‐CO electrocatalysts, as extracted from the DigCat Database (https://www.digcat.org/, the largest experimental electrocatalysis database to date). For easier comparison, the maximum *J*_CO_ and FE_CO_ values are extracted and displayed in Figure 3d (left). These results show that our spray‐grown CoPc crystals outperform all previously reported M‐Pc catalysts.^[^ ^18^, ^25^, ^26^, ^30^, ^31^, ^32^, ^33^, ^34^, ^35^ ^]^ Notably, previous studies have indicated that industrial applications require a *J*_partical_ exceeding 500 mA cm^−2^ and an FE > 98%. Only our catalyst meets these industrial requirements, while previously reported catalysts struggled to reach this threshold of −500 mA cm^−2^. Further comparisons of key parameters, including turnover frequency (TOF) and mass activity (MA), are also provided in Table 1 and visually represented in the radar plots (Figure 3d, right). Specifically, our catalyst demonstrated the highest MA of 5180 A g⁻¹, compared to a maximum of 1000 A g⁻¹ reported in ref. [25]—approximately five times higher, indicating the superior conversion efficiency of our spray‐grown CoPc per unit mass (see Table S4, Supporting Information for more details). Additionally, our catalyst achieved a TOF of 13.8 s⁻¹, compared to the highest reported TOF of 9.8 s⁻¹ from ref. [18], highlighting the high catalytic efficiency at each active site. Therefore, the spray‐grown CoPc/CP developed in this study demonstrates significant potential for industrial applications.

**Table 1:** Comparison of the key values in this work and literature.

Catalyst	pH	FE_CO_ [%]	*J*_CO_ [mA cm^−2^]	TOF [s^−1^]	MA [A g^−1^]	Stability @ *J* [hour @ mA cm^−2^]	Refs.
CoPc crystals	14.0	>98.0	−1036	13.8	5180	145 @ −150	This work^*^
CoPc/CNT	6.8	98.0	−15	4.1	37.5	10 @ −10^a^ ^)^	[25]
CoPc/CNT	2.0	73.0	−38	–	47.5	27 @ −90^a^ ^)^	[38]
CoPc/CB	7.8	98.0	−100	–	–	10 @ −3^a^ ^)^	[34]
CoPc/CNT‐MDE	6.8	97.0	−200	–	–	38 @ −250	[32]
CoPc/CNT‐ODA	7.3	97.7	−350	–	350	12 @ −150^a)^	[33]
CoPc‐TBG/CNT	14.0	96.0	−112	2.7	560	3 @ −150	[35]
CoPc‐ EtO_8_/CNP	7.8	95.0	−340	–	–	24 @ −150	[30]
CoPc‐OCH_3_/CNT	7.3	97.0	−280	9.8	–	10 @ −150	[39]
CoPPc/CNT	7.3	90.0	−19	1.25	19	24 @ −15	[40]
NiPc/CNT‐MDE	7.3	> 98.0	−400	12.0	1000	40 @ −150	[18]
NiPc/NHCSs	7.3	98.6	−25	–	25	32 @ −100	[41]
NiPc(OH)_6_(DCNFO)/CNT	14.0	> 98.0	−380	3.3	–	40 @ −150	[31]
NiPc‐OMe	2.0	> 98.0	−400	–	400	12 @ −100	[29]

^a)^ (the experiments were conducted under constant voltage, so the *J* values are estimated from the corresponding *J*‐*t* curves).

Besides these metrics, electrochemical stability is also a crucial factor in assessing the industrial potential of catalysts. Long‐term stability tests on our CoPc/CP were conducted at a *J* of −150 mA cm^−2^. As shown in Figure 3c, the potential remained stable at ≈−0.55 V (vs RHE) over 145 h (6 days) of continuous operation, with neglected degradation and a consistently high FE_CO_ above 95%. Detailed electrolysis times and their corresponding applied *J* values from the literature are also listed in Table 1 and vividly compared in Figure 3d (right). Notably, the longest reported stability is only 40 h at a *J* of −150 mA cm^−2^, as obtained by ref. [18]. We propose that the excellent stability can be attributed to the following reasons: unlike traditional carbon‐supported methods that rely on adsorption, spray‐grown CoPc crystals exhibit stronger substrate adhesion, ensuring structural stability during long‐term electrolysis. Furthermore, even if surface active sites detach, the inner catalyst effectively replenishes them, maintaining overall activity. These structural features are unique to our spray‐grown electrodes and are absent in traditional carbon‐based electrodes.

Interestingly, all five key parameters shown in the radar plots are derived from five previously reported catalysts. However, our catalyst displayed a larger radar plot area, indicating superior overall performance compared to these benchmarks. This suggests that our catalyst combines all high‐performance characteristics in one. Overall, these results demonstrate that our spray‐grown electrodes not only exhibit excellent electrocatalytic activity but also remarkable long‐term stability, highlighting their potential as promising ECR electrocatalysts for industrial applications.

### 2.4 Mechanism Investigation

**Figure 4:**
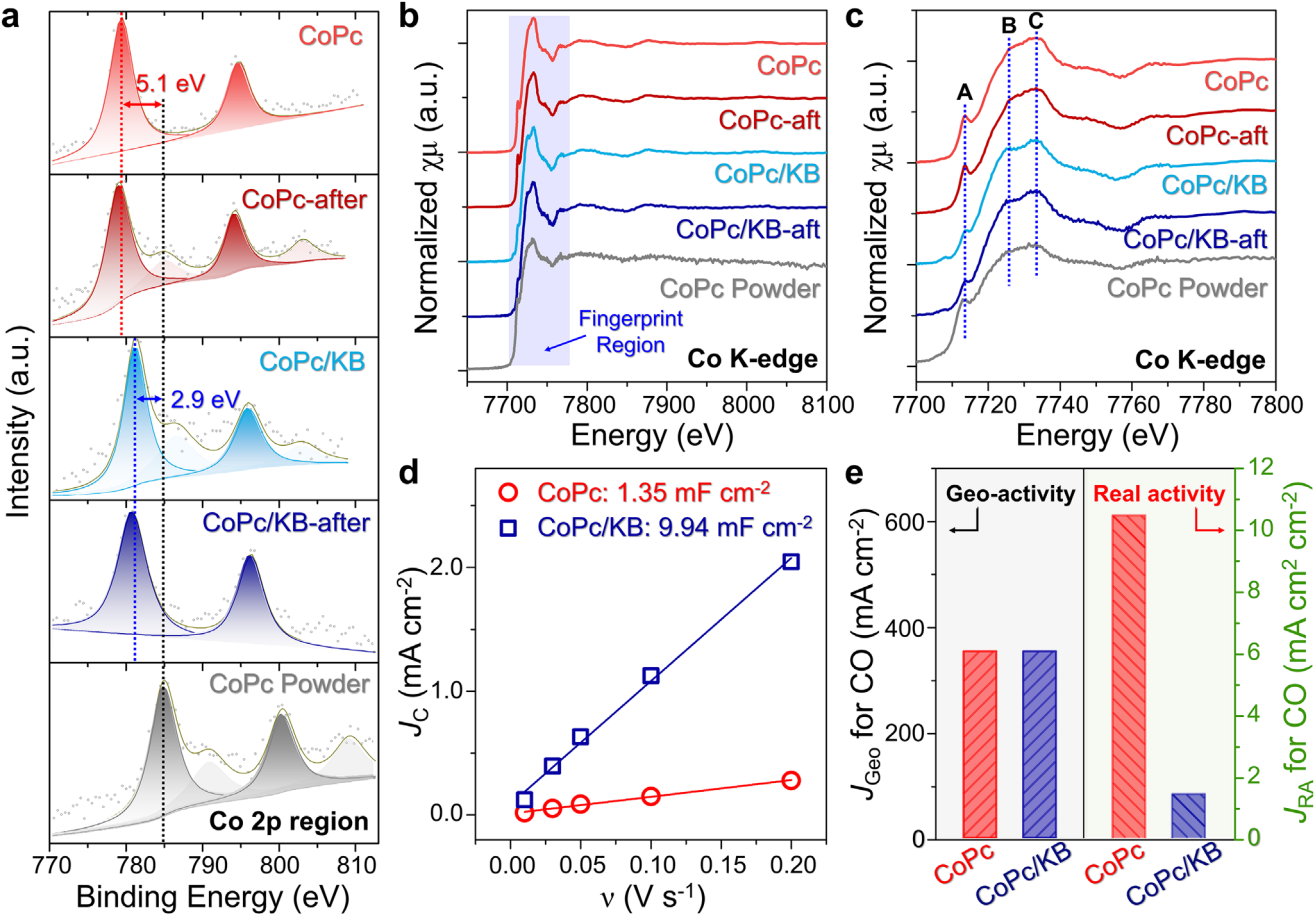
Origin of high performance in CoPc crystals for ECR. a) XPS spectra of the Co 2p region, b) XANES spectra at the Co K‐edge, and c) extended fingerprint region, for CoPc/CP before ECR (pink) and after ECR (dark red), CoPc/KB/CP before ECR (cyan) and after ECR (royal), and CoPc powder (gray). d) Double‐layer capacitance (*C*_DL_) derived from fitting the capacitance current density (*j*_C_) against scan rate (*v*) for CoPc/CP and CoPc/KB/CP electrodes. e) Comparison of geometric activity (left axis) and real activity (right axis) for CoPc/CP and CoPc/KB/CP electrodes, based on geometric current density (*J*_Geo_) and current density normalized by electrochemical surface area (*J*_RA_) for CO at a potential of −0.94 V (vs. RHE) in 1.0 m KOH.

These electrochemical results demonstrate that the spray‐grown CoPc electrode offers notably high performance, underscoring its strong potential for industrial applications. Cross‐sectional SEM and elemental mapping images (Figures S6 and S7, Supporting Information) confirm the retention of CoPc crystals on the CP electrode even after electrolysis. Elucidating the mechanisms responsible for this exceptional performance is critical for extending the method to enhance other M–Pc materials for ECR. As previously discussed, structural characterization shows that CoPc crystals preferentially grow along the (−102) plane during the spray‐growth process, implying a robust interaction between the CoPc crystals and the CP substrate. This interaction likely enhances substrate adhesion, supporting long‐term stability, and facilitates electron transfer between the substrate and the catalyst. X‐ray photoelectron spectroscopy (XPS) analysis showed a 5.1 eV negative shift in the Co 2p binding energy of the spray‐grown CoPc crystals relative to CoPc powder (**Figure** **4**a), indicating successful electron transfer to the Co atoms via layer‐by‐layer charge transfer. In contrast, CoPc supported on Ketjen‐black (CoPc/KB) exhibited only a 2.9 eV shift, consistent with reports that the high surface area of KB disperses and stabilizes phthalocyanine molecules, thereby limiting crystal growth. To validate this charge‐induced shift, we also performed XPS analysis in the C 1s region (Figure S8, Supporting Information), where signals from carbon species were derived from both the organic macrocycle and carbon substrates. Notably, the phthalocyanine‐related peaks in the spectra of CoPc/CP, CoPc/KB, and CoPc powder were located at the same binding energy without any shift, confirming the reliability of the shift observed in the Co 2p region.

Based on these results, we propose that the spray‐grown CoPc possesses a unique crystalline structure and strong interaction with the substrate, leading to improved charge transfer capability. Additionally, the larger crystal structure compared to CoPc/KB allows for greater surface charge transfer, which may be a key factor contributing to its enhanced electrocatalytic activity.

While XPS primarily provides information on surface properties, XANES offers a broader view of the catalyst's overall characteristics due to the penetrating nature of X‐rays. XANES analysis showed that CoPc crystals, CoPc/KB, and CoPc powder exhibit similar spectral features (Figure 4b), indicating no significant differences in their bulk structures. However, closer inspection of the fingerprint region revealed slight variations in peaks at positions A, B, and C, which are likely associated with differences in the extent of charge transfer (Figure 4c). The EXAFS spectra also displayed similar features across all samples (Figure S9, Supporting Information). These findings further confirm that charge transfer primarily occurs at or near the surface of CoPc crystals, which constitutes only a small portion of the overall crystal structure.

Notably, this charge transfer phenomenon endures even after the CO_2_RR process, indicating that the charge‐enriched CoPc crystals remain actively involved throughout the reaction. Based on these observations, we propose that spray‐grown CoPc crystals establish robust interactions with the carbon substrate, substantially enhancing spontaneous surface charge transfer compared to traditional carbon‐loaded CoPc. This sustained surface charge transfer amplifies the intrinsic activity of Co‐N_4_ active centers on the CoPc crystal surface, significantly enhancing the overall electrocatalytic efficiency. Moreover, additional electrochemical data further demonstrated the superior activity of CoPc crystals compared to CoPc/KB across various loadings and potentials (Figure S10, Supporting Information).

To further validate this hypothesis, we performed cyclic voltammetry (CV) scans on CoPc crystals and CoPc/KB (Figure S11, Supporting Information), calculating the double‐layer capacitance (*C*_dl_) at varying scan rates to determine the electrochemical active surface area (ECSA).^[^ ^27^, ^36^, ^37^ ^]^ The results indicated that the *C*_dl_ values for CoPc crystals and CoPc/KB were 1.35 and 9.94 mF cm^−2^, respectively, corresponding to ECSAs of 33.75 and 248.5 cm^2^ cm^−2^ (Figure 4d). These findings clearly show that the incorporation of KB significantly increases the ECSA.

Notably, despite the larger ECSA of CoPc/KB, both CoPc crystals and CoPc/KB exhibited nearly identical geometric current densities (*J*_Geo_) at the same voltage, ≈350 mA cm^−2^. This indicates that the active sites on CoPc crystals are more efficient. While ECSA indicates the number of active sites, the current density for CO_2_ reduction to CO represents the total electron transfer, reflecting the overall reaction efficiency. Thus, dividing *J*_Geo_ by ECSA yields the true efficiency of individual active sites, defined as the real active current density (*J*_RA_). Area‐normalized calculations reveal that the *J*_RA_ values of CoPc crystals and CoPc/KB are 10.5 and 1.4 mA cm^−2^ cm^2^, respectively, indicating that the efficiency of each active site on CoPc crystals is 7.6 folds that of CoPc/KB. These findings further confirm that surface charge transfer greatly enhances the intrinsic electrocatalytic activity of crystalline CoPc active sites. Furthermore, we also conducted in situ electrochemical impedance spectroscopy (EIS) measurements for CoPc crystals under a CO_2_ atmosphere at different potentials (Figure S12, Supporting Information). The charge transfer resistance (R_ct_) values at *E* = −0.83, −0.94, and −1.04 V vs RHE were 0.76, 0.22, and 0.13 Ω, respectively, indicating that R_ct_ decreases with more negative potentials, facilitating easier charge transfer. For CoPc/KB, the R_ct_ values were 1.05, 0.38, and 0.24 Ω at the same potentials, demonstrating that CoPc crystals exhibit lower R_ct_ and superior charge transfer efficiency.

**Figure 5:**
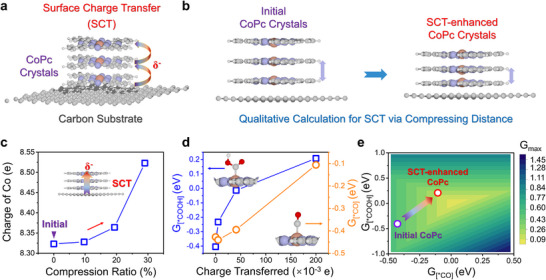
Origin of surface charge transfer (SCT) enhancement in CoPc crystals. a) Multilayer model simulating CoPc crystals grown on the carbon substrate. b) Qualitative calculation for SCT in CoPc crystals by compressing interlayer distance. c) Bader charge variation of Co atoms with interlayer compression. d) Adsorption free energies (G_[*COOH]_ and G_[*CO]_) as a function of charge transferred at Co sites. e) Volcano model with adsorption free energies (G_[*COOH]_ and G_[*CO]_) for initial and SCT‐enhanced CoPc crystals, indicating SCT‐driven high intrinsic activity, consistent with experimental results.

Electrochemical data and characterization analyses underscore the pivotal role of surface charge transfer in enhancing catalytic performance. Further analyses conclusively confirm that the observed CO is derived from CO_2_ feedstocks, ruling out the possibility that it originates from the carbon substrate (Figure S13, Supporting Information). To clarify how surface charge transfer operates, we performed DFT calculations on the CoPc/carbon model, placing the intermediates directly above the Co center along the axis perpendicular to the molecular plane. First, we developed a multilayer model to represent CoPc crystals grown on the carbon substrate (**Figure** **5**a), systematically reducing the interlayer distance to generate negatively charged environments around the Co centers (Figure 5b). By gradually compressing the interlayer distance from 3.1 to 2.2 Å (Figure S14, Supporting Information), we could effectively calculate surface charge transfer within the CoPc crystals. Bader charge analysis provided a quantitative visualization of electron transfer as interlayer compression increased (Figure 5c). We further assessed the impact of this charge transfer on intrinsic catalytic activity by calculating the adsorption free energies of ^*^COOH and ^*^CO (G_[*COOH]_ and G_[*CO]_) (Figure 5d). As charge transfer increased, the adsorption free energies rose noticeably, indicating a weakening interaction between these intermediates and the outermost Co sites. When mapped on the Volcano model with adsorption free energies (Figure 5e), these results vividly demonstrated that surface charge transfer significantly enhanced the intrinsic activity of catalytic sites, bringing the CoPc system closer to peak performance. Taken together, our findings provide solid evidence that spray‐grown CoPc crystals exhibit self‐induced surface charge transfer, greatly boosting the intrinsic activity for the electrochemical conversion of CO_2_ to CO.

## 3 Conclusion

In this study, we developed a spray‐growth method to rapidly load CoPc crystals onto carbon paper, achieving exceptional CO_2_‐to‐CO electroreduction performance in alkaline electrolytes. The electrode demonstrated over 90% CO selectivity across a broad electrochemical window (−0.57 to −1.32 V vs RHE) and a record *J*_CO_ of −1036 mA cm^−2^ at −1.32 V (vs RHE), with the ultrahigh mass activity of 5180 A g⁻¹, outperforming all previously reported M‐Pc catalysts. Additionally, it maintained a stable potential of −0.55 V (vs RHE) for 145 h at −150 mA cm^−2^, showcasing excellent long‐term stability. These spray‐grown CoPc catalysts exhibited a 7.6‐fold improvement in real activity compared to CoPc/KB, indicating enhanced intrinsic activity. Structural characterization and DFT calculations attribute this remarkable performance to the unique crystalline structure and spontaneous surface charge transfer (SCT), which generates negatively charged Co sites and optimizes binding energies, significantly boosting Co‐N_4_ site activity and enhancing both efficiency and stability. Unlike traditional carbon‐supported methods, spray‐grown CoPc crystals demonstrated stronger substrate adhesion, ensuring structural integrity during long‐term electrolysis, with the inner catalyst replenishing detached surface sites. We believe this simple and scalable spray‐growth method holds broad applicability for producing phthalocyanine‐based materials, with the potential to elevate the role of M‐Pc materials in ECR and contribute to carbon neutrality.

## Conflict of Interest

The authors declare no conflict of interest.

## Author Contributions

T.L. and D.Z. authors contributed equally to this work. T.L. conducted conceptualization, investigation, methodology, resources, formal analysis, and writing of the original draft. D.Z. handled conceptualization, methodology, and writing of the original draft. Y.H., K. I., K. I., N.T., Y.M., J.Y., and S.O. participated in methodology, resources, and formal analysis. H.L. and H.Y. led conceptualization, resources, writing—review and editing, and supervision. T.L., H.L., and H.Y. coordinated collaboration among all co‐authors and finalized the draft. All authors contributed to the revisions.

## Supporting information



Supporting Information

## Data Availability

The data that support the findings of this study are available from the corresponding author upon reasonable request.
