# Supplementary material for: Surface Charge Transfer Enhanced Cobalt‐Phthalocyanine Crystals for Efficient CO2‐to‐CO Electroreduction with Large Current Density Exceeding 1000 mA cm−2
Source: Adv Sci (Weinh). 2025 Apr 4;12(23):2501459. doi: 10.1002/advs.202501459 (PMC12199440; doi:10.1002/advs.202501459)
Supplement: Supplementary file 1 — Supporting Information [file ADVS-12-2501459-s001.docx]

Supporting Information

**Surface Charge Transfer Enhanced Cobalt-Phthalocyanine Crystals for Efficient CO_2_-to-CO Electroreduction with Large Current Density Exceeding 1000 mA cm^-2^**

*Tengyi Liu,*^†,*^ *Di Zhang,*^†^ *Yutaro Hirai, Koju Ito, Kosuke Ishibashi, Naoto Todoroki, Yasutaka Matsuo, Junya Yoshida, Shimpei Ono,* *Hao Li,*^*^ *and Hiroshi Yabu*^*^

**S-1. Experiment Section**

**S-1.1. Materials and Regents：**

Non-metal phthalocyanine (H_2_Pc) and metal phthalocyanines (M-Pcs), including FePc, CoPc, NiPc, and CuPc, were supplied by Azul Energy Corporation, each with a purity higher than 99.9%. Ketjen-Black carbon black (EC-600JD) was sourced from Fuel Cell Store. 2-Propanol (IPA) and dimethyl sulfoxide (DMSO) were obtained from Fuji-Wako, each with a purity above 99.5%. Nafion dispersion solution (20 wt%) was purchased from Sigma-Aldrich. The carbon paper gas diffusion layers used for electrochemical CO_2_ reduction tests were acquired from Mitsubishi Chemical Corporation (PYROFIL-GDL MFK-A, 0.21 mm), while those (ABOUND-GDL) for long-term durability tests were supplied by Abound Energy Corporation. All gases, including carbon dioxide (CO_2_), carbon monoxide (CO), methane (CH_4_), ethylene (C_2_H_4_), and hydrogen (H_2_), were purchased from Taiyo Nippon Sanso Company, each with a purity over 99.9%. Ultrapure water (18.2 MΩ) was obtained through a purification system. All reagents were used as received without further purification.

**S-1.2 Equipment and Devices:**
 The following equipment was used in this study. A ball-mill (FRITSCH, Planetary Mono Mill Pulverisette 6) ground materials into fine powder, while a sim-coat system (Sono-Tek) applied uniform catalyst films onto substrates. Gas chromatography with flame ionization detection (GC-FID, Shimadzu GC-2014) analyzed gaseous products, and an electrochemical workstation (Princeton Applied Research, Versa-STAT 4) conducted electrochemical measurements, including CO_2_ reduction tests. An analytical balance (A&D, BM-20) ensured precise mass measurements, and an oven (Espec, ST-120) was used for controlled drying. An ultrasonic machine (Asone, ASU-2M) dispersed inks, and gas flow meters (FCON, C2005-CO_2_-003) monitored gas flows. Ultrapure water was generated using an ELGA (LA-758 Purelab system) purification system. X-ray photoelectron spectroscopy (XPS, Ulvac-PHI 5000 Versa Probe II) analyzed surface composition, X-ray diffraction (XRD, RIGAKU Smart-Lab) determined crystalline structures, and X-ray absorption fine structure (XAFS, SPring-8 & NanoTerasu) spectroscopy provided local electronic insights. A scanning electron microscope (SEM, JEOL JSM-7800F) and field emission electron probe microanalysis (FE-EPMA, JEOL JXA-8530F) were used for imaging and elemental analysis.

**S-1.3. Preparation of Relative Electrodes：**
**S-1.3.1. M-Pc crystals grown on carbon paper (M-Pc/CP) electrode:**

In this work, we selected H_2_Pc and four key M-Pcs (FePc, CoPc, NiPc, and CuPc) to prepare the respective electrodes, the corresponding chemical structures are shown in **Figure S1**. The process for fabricating CoPc/CP GDE is presented as an example, and shown as follows: First, CoPc powder (e.g., 100 mg) was measured using an analytical balance and placed into a ball-mill autoclave with 100 g balls of ZrO₂. IPA (9 mL), DMSO (9 mL), ultrapure water (4.5 mL), and Nafion dispersion (20 wt%, 0.2 mL, or 0.1 mL per 50 mg catalyst) were then added. The mixture was ball-milled for 5 minutes to form the ink precursor. After milling, the autoclave and balls were cleaned with a solution of IPA (45 mL), DMSO (9 mL), and ultrapure water (13.5 mL). The resultant ink, containing totally 100 mg catalyst and 90 mL solvent, was ultrasonically dispersed for 5 minutes for uniformity. It was then spray-coated onto pre-cut carbon paper on a heated plate (70°C) to evaporate the solvent and promote CoPc crystal growth. It is important to note that the ball-milling process is a purely physical procedure that does not alter the molecular structure of M-Pc. Additionally, the rapid evaporation of the solvent at high temperatures promotes the orderly alignment of CoPc molecules along the carbon paper substrate. Catalyst loading was calculated by dividing the mass of deposited CoPc by the surface area (6 × 6 cm²), determined by the weight difference before and after spray-coating. The loading was controlled by adjusting the ink concentration or varying the number of spray coatings, with the latter being the primary method used in this study. In this work, the optimal CoPc/CP electrode, with a loading of approximately 0.2 mg/cm², was prepared using 100 mg of catalyst in 90 mL solution and applying about 8~12 spray-coating layers.

**S-1.3.2. Carbon-support M-Pcs electrode:**

In this work, traditional carbon-supported M-Pc catalysts were also prepared as a reference. As an example, 20 wt% CoPc supported on Ketjen Black (KB) carbon black was synthesized. CoPc (200 mg) and KB (800 mg) were mixed in a round-bottom flask with DMSO (150 mL) and sonicated in an ice-water bath for 30 minutes to allow molecular adsorption of CoPc onto KB.^[1]^ The mixture was then filtered and washed three times with methanol. The resulting cake was dried at a moderate temperature (e.g., 35°C) in a vacuum oven for 24 hours to avoid activity loss. The dried hybrid was ground in a mortar, weighed, and placed into a ball-mill autoclave. The solvent and ratios were the same as those used for CoPc crystals, but the ball milling time was extended to at least 30 minutes due to the strong agglomeration of the carbon-based hybrid. Ultrasonic dispersion was also increased to 30 minutes. The resulting ink was spray-coated onto carbon paper to form the CoPc/KB hybrid electrode. Various loadings were prepared by adjusting ink concentration and spray-coating numbers. Typically, 500 mg of CoPc/KB in 90 mL solution corresponds to 100 mg of CoPc ink, yielding similar loadings of CoPc with the same spray-coating number.

**S-1.4. Electrochemical CO_2_ Reduction (ECR) Test:**

The prepared electrode was cut and used as the working electrode in a custom three-electrode electrolyzer, with Hg/HgO (1.0 M KOH) as the reference electrode and Pt wire as the counter electrode, the detailed components and main equipment are shown in **Figure S1**. The 1.0 M KOH solution served as the electrolyte. The carbon-based gas diffusion electrode (GDE) was masked with a tape ring (0.5 cm² hole) and used as the cathode, with CO_2_ fed from the back of the GDE, and the flow controlled by a flow meter (e.g., 15 sccm). This setup optimizes the solid-liquid-gas interface and prevents electrode flooding. The anode and cathode chambers were separated by a Nafion-117 membrane, which was chosen to avoid the transfer of ECR products into the anode, as this could cause errors, even though an anion exchange membrane may offer higher exchange ratios.^[2]^ After connecting the setup to the electrochemical workstation, gas products were collected in a gas bag, and their volume was measured using a flow meter. The gases were analyzed by GC-FID and selectivity and partial current density were obtained (details in the next section). The applied potentials were adjusted to optimize faradaic efficiency and current density for CO_2_ reduction. The potential vs. reference was converted to the reversible hydrogen electrode (RHE) using the equation:

*E* (vs. RHE) = *E* (vs. Ref.) + 0.0592 × pH + *E*₀ (Ref.) - *iR*

Where *E* (vs. Ref.) is the applied potential, *E*₀ (Ref.) is the standard potential of the reference electrode (0.105 V for Hg/HgO in 1.0 M KOH),^[3]^ and *iR* is the ohmic drop.

For standard ECR tests, electrolysis was performed on an MFK-A GDE for about 900 seconds using 25 mL KOH electrolyte in both the cathode and anode chambers. For long-term durability tests, liquid pumps were used to refresh the anolyte and catholyte, and ABOUND-GDL was employed to prevent flooding.

**S-1.5. Qualitative and Quantitative Analysis of ECR Products:**

In this work, we use gas chromatography with a flame ionization detector (GC-FID) to analyze the ECR products. Gases displayed different peak positions in real-time based on molar mass, with lighter gases appearing earlier and heavier gases later in the intensity vs. time pattern. The main ECR products included H_2_, CH_4_, CO, and C_2_H_4_. The first three gases were detected using a thermal conductivity detector (TCD), while C_2_H_4_ was detected only by FID, likely due to FID's sensitivity to hydrocarbons. Following the principles of GC, H_2_, CH_4_, and CO eluted in sequence with retention times around 0.7, 3.0, and 5.9 minutes, respectively. Slight variations in peak times occurred due to carrier gas flow rates but did not affect the overall peak order, enabling reliable qualitative analysis.

Peak area in the intensity vs. time graph is proportional to gas concentration. Calibration lines were created for four gases by preparing standard mixtures. For example, a 20% CO standard was made by mixing 80 mL of CO_2_ with 20 mL of CO in a sample bag and analyzing it with GC-FID. Each sample was measured three times for accuracy. Calibration curves showed R² values over 0.99, indicating high reliability. The detailed ratios of mixtures, peak areas and relative curves are presented in **Figure S2,** **Table S1** and **Figure** **S3**, respectively.

During ECR electrolysis, gas products were collected in sample bags, and total volume was recorded. Gas content and peak areas were analyzed with GC-FID, and gas ratios were determined using the calibration curves. The detailed ECR system and electrolyzer are shown in **Scheme S1**. From this, the molar quantities of each gas were calculated. For example, CO moles (n_CO_) were determined from peak areas. Using the CO_2_-to-CO reaction stoichiometry, the electron transfer for CO production was calculated with Q_CO_ = n_CO_ × F × N_CO_, where F is Faraday's constant (96485 C/mol), and N_CO_ is the number of electrons transferred (N_CO_ = 2). The chemical reactions and corresponding numbers of electrons transferred for each reaction are shown in **Table S2**.^[4,5]^

The total charge (Q_total_) was determined by Q_total_ = I × t, where I is the current recorded by the electrochemical workstation, and t is the relative time. Faradaic efficiency for CO (FE_CO_) was calculated as FE_CO_ = Q_CO_ / Q_total_. Finally, the total current density (*J*_total_) was calculated by dividing the current by the electrode surface area (0.5 cm²), and the partial current density for CO (*J*_CO_) was determined using *J*_CO_ = *J*_total_ × FE_CO_.

**S-1.6. Computational methods：**

In this study, we performed density functional theory (DFT) calculations using the Vienna Ab initio Simulation Package (VASP) to investigate the electronic and magnetic properties of the system. The generalized gradient approximation (GGA) with the RPBE functional was employed to describe the exchange-correlation effects.^[6–8]^ The plane-wave basis set energy cutoff (ENCUT) was set to 520 eV to ensure accurate results. Spin-polarized calculations were performed, allowing for the consideration of magnetic effects. The electronic convergence criterion was set to 1 × 10^-6^ eV, while the convergence criterion for ionic relaxation was set to −0.05 eV/Å. We utilized the DFT-D3 method with Becke-Johnson damping to account for van der Waals interactions, ensuring that dispersion forces were included.^[8,9]^

Adsorbate binding and free energies were calculated with the lowest energy adsorbate configuration. The binding energies of CO and COOH are, respectively,

$$E_{b}(CO)=E(*CO)+\Delta E_{sol}(CO)-E*-E(CO_{2})-E(H_{2})+E(H_{2}O)$$

$$E_{b}(COOH)=E(*COOH)+\Delta E_{sol}(COOH)-E*-E(CO_{2})-\frac{1}{2}E(H_{2})$$

Where ΔE_sol_ indicates a solvation correction to the calculated adsorbate energy. ΔE_sol_(COOH)=−0.25 eV and ΔE_sol_(CO) = −0.1 eV.^[10]^ The free energies (G) of the adsorbate states were calculated by incorporating zero-point energies, entropies, and heat capacities, as outlined in the reference.^[10]^ The activity volcano plots were developed by our previous work.^[11]^ All the multilayered CoPc structures are available in the GitHub https://github.com/tohokudizhang/CoPc_ChargeTransfer.


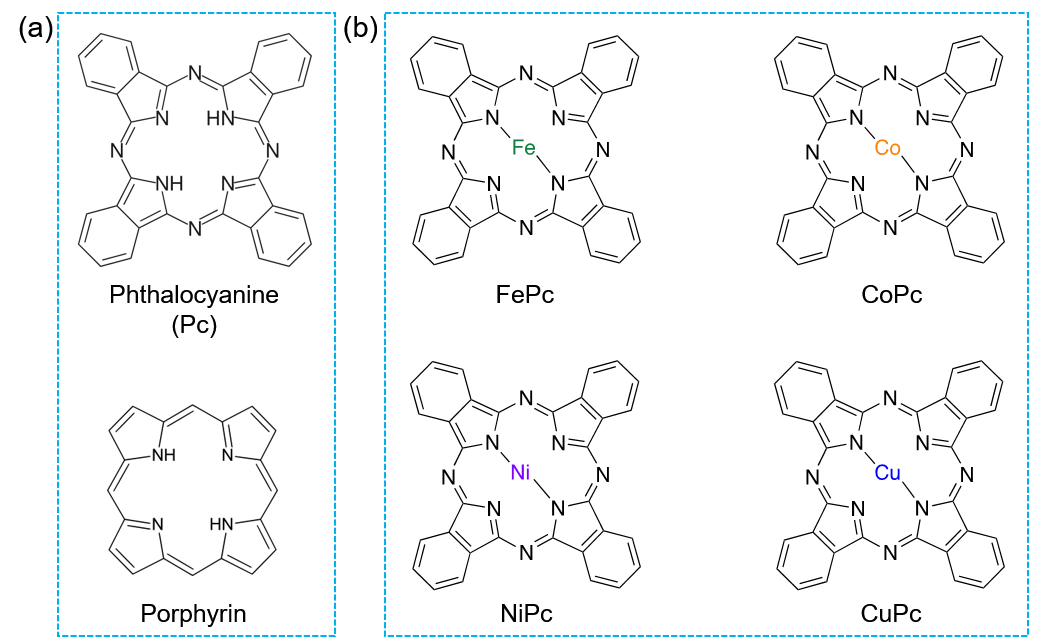


**Figure S1.** The chemical structures of various molecular materials: (a) Non-metal phthalocyanine (top) and porphyrin (bottom), (b) four typical metal phthalocyanines.


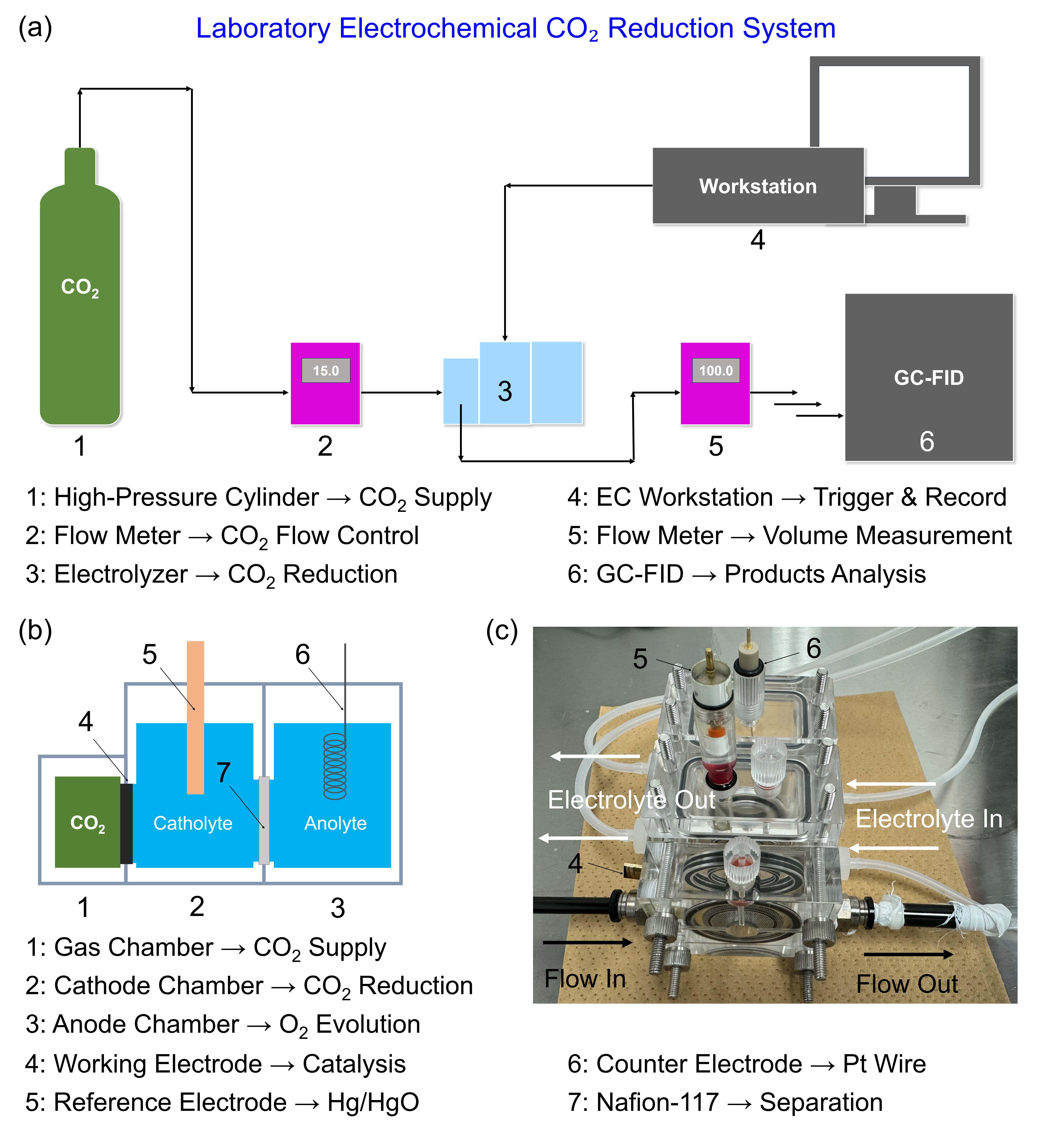


**Scheme S1.** Laboratory electrochemical CO_2_ reduction (ECR) system: (a) main components and equipment of the laboratory ECR system; (b) schematic diagram, and (c) actual image of the custom-made three-electrode electrolyzer.


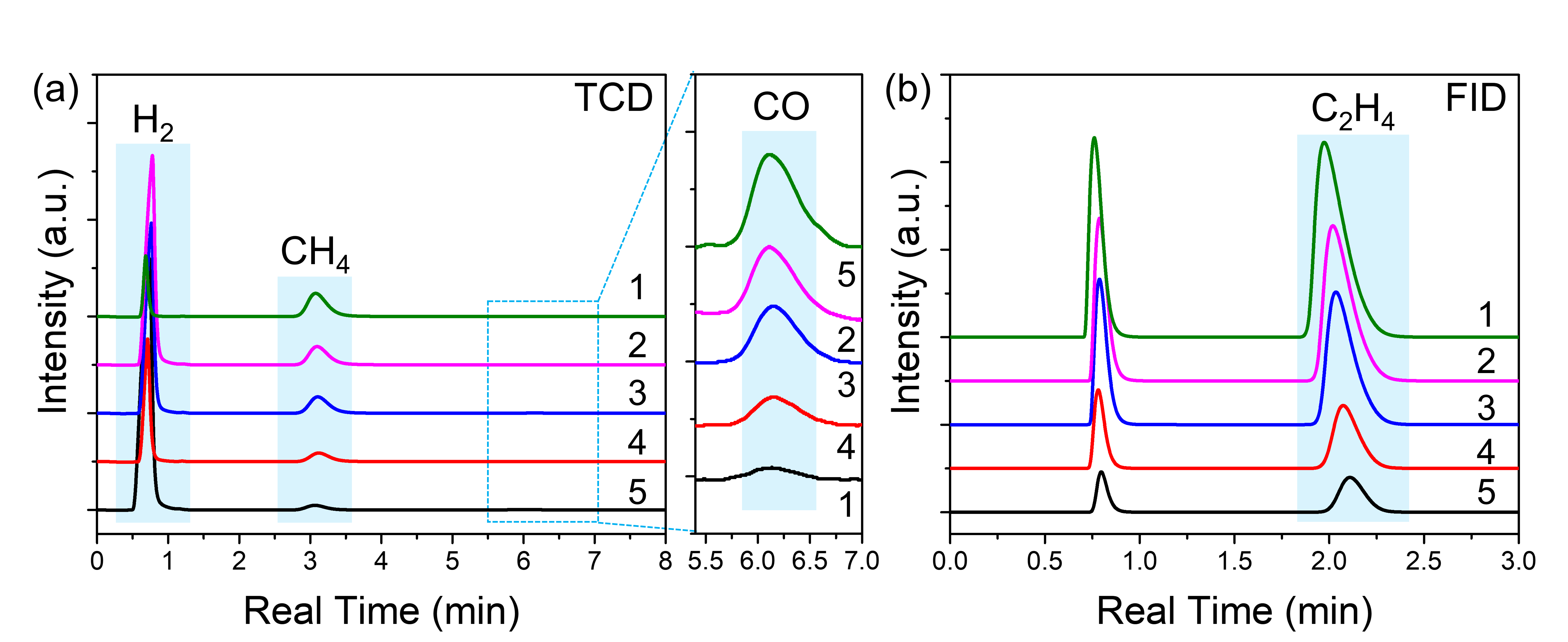


**Figure S2.** GC-FID measurements for five gas mixtures with varying ratios of C_2_H_4_, CH_4_, CO, H_2_, and CO_2_ for calibration lines. (a) TCD patterns for H_2_, CH_4_, and CO (inset); (b) FID patterns for C_2_H_4_. The detailed mixture compositions are shown in Table S1.

**Table S1.** Five gas mixtures of four target gases (C_2_H_4_, CH_4_, CO, and H_2_) with varying ratios in CO_2_, with a total volume of 100 mL. The peak areas were detected by GC-FID, derived from Figure S1, and correspond to their respective ratios.

| **Mixture**  **(100 mL)** | **C_2_H_4_**  **(%)** | **Peak area (C_2_H_4_)** | **CH_4_**  **(%)** | **Peak area (CH_4_)** | **CO**  **(%)** | **Peak area (CO)** | **H_2_**  **(%)** | **Peak area (H_2_)** |
| --- | --- | --- | --- | --- | --- | --- | --- | --- |
| **1** | 35 | 4966457704 | 35 | 94961.9 | 5 | 317.1 | 5 | 59966.3 |
| **2** | 25 | 3723620419 | 25 | 74221.1 | 25 | 1626.8 | 25 | 380926 |
| **3** | 20 | 3086653626 | 20 | 66997.2 | 20 | 1242.2 | 20 | 315930.7 |
| **4** | 10 | 1400624225 | 10 | 35227 | 10 | 745.9 | 10 | 153834 |
| **5** | 5 | 760868344 | 5 | 18439.6 | 35 | 2309.27 | 35 | 541309.3 |


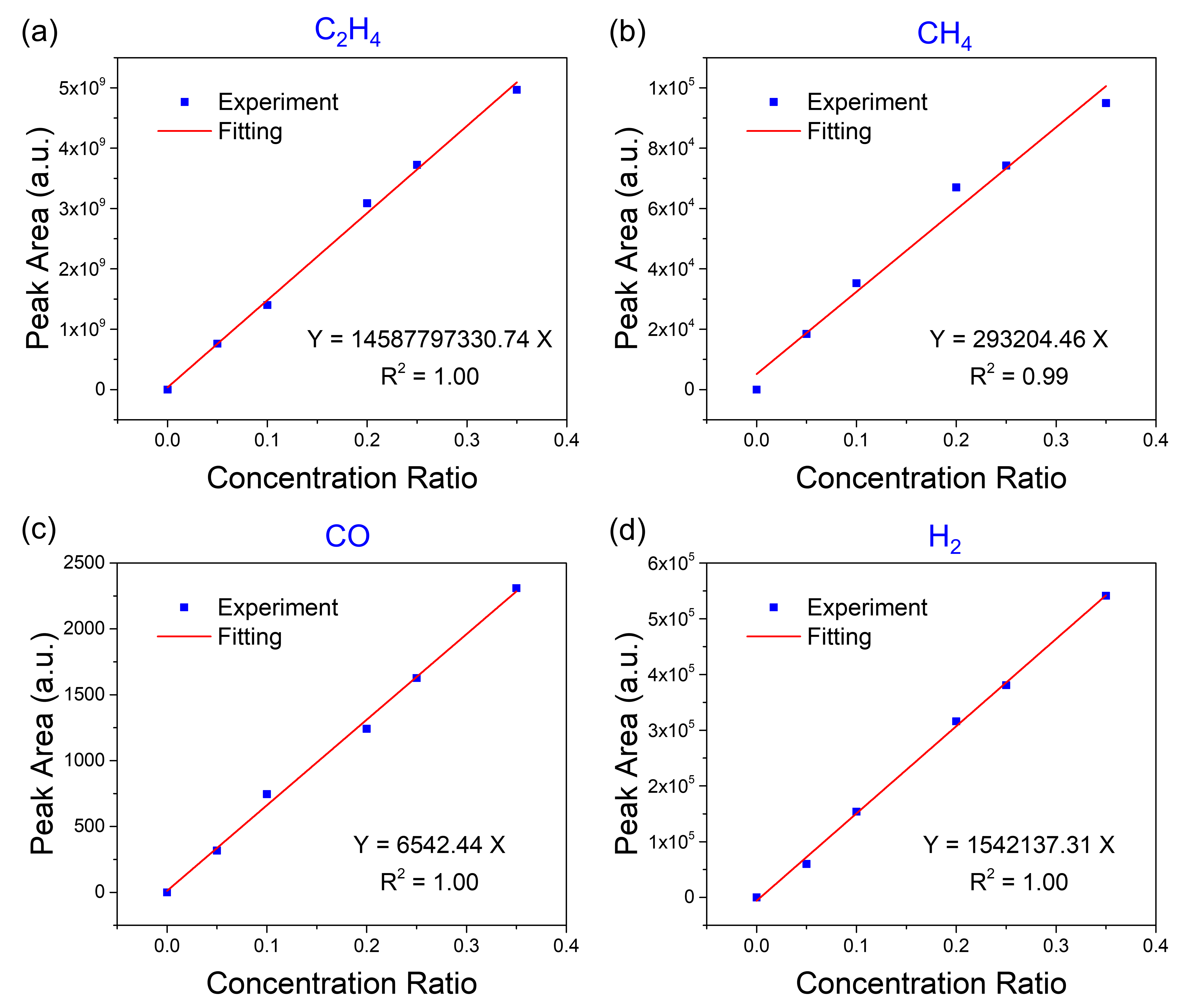
 **Figure S3.** Calibration lines for the four target gases: (a) C_2_H_4_, (b) CH_4_, (c) CO, and (d) H_2_, at varying ratios. Peak areas and concentration ratios are derived from Figure S1 and Table S1. Note: e.g., a concentration ratio of 0.1 corresponds to a 10% volume ratio.

**Table S2.** The chemical reactions and corresponding numbers of electrons transferred for each reaction.^[4,5]^

| **Product** | **N** | **Half Reaction (pH =14)** | ***E*_0_ (vs. RHE)** |
| --- | --- | --- | --- |
| **H_2_** | 2 | 2H_2_O (l) + 2e^−^ → H_2_ (g) + 2OH^−^ | 0 |
| **CO** | 2 | CO_2_ (g) + H_2_O (l) + 2e^−^ → CO (g) + 2OH^−^ | -0.106 |
| **CH_4_** | 8 | CO_2_ (g) + 6H_2_O (l) + 8e^−^ → CH_4_ (g) + 8OH^−^ | 0.164 |
| **C_2_H_4_** | 12 | 2CO_2_ (g) + 8H_2_O (l) + 12e^−^ → C_2_H_4_ (g) + 12OH^−^ | 0.074 |


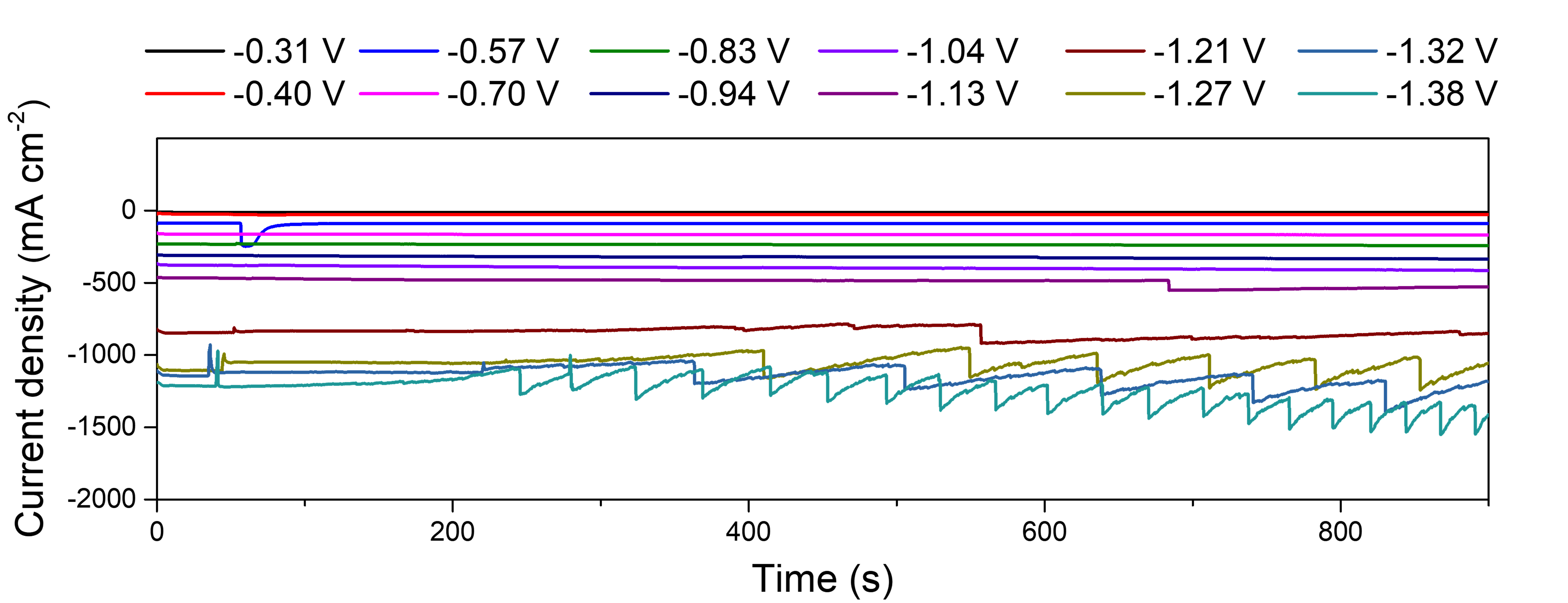


**Figure S4.** Time-dependent total current density curves at various applied potentials for CO_2_ reduction using the CoPc/CP electrode with a loading of ~0.2 mg/cm² in 1.0 M KOH.


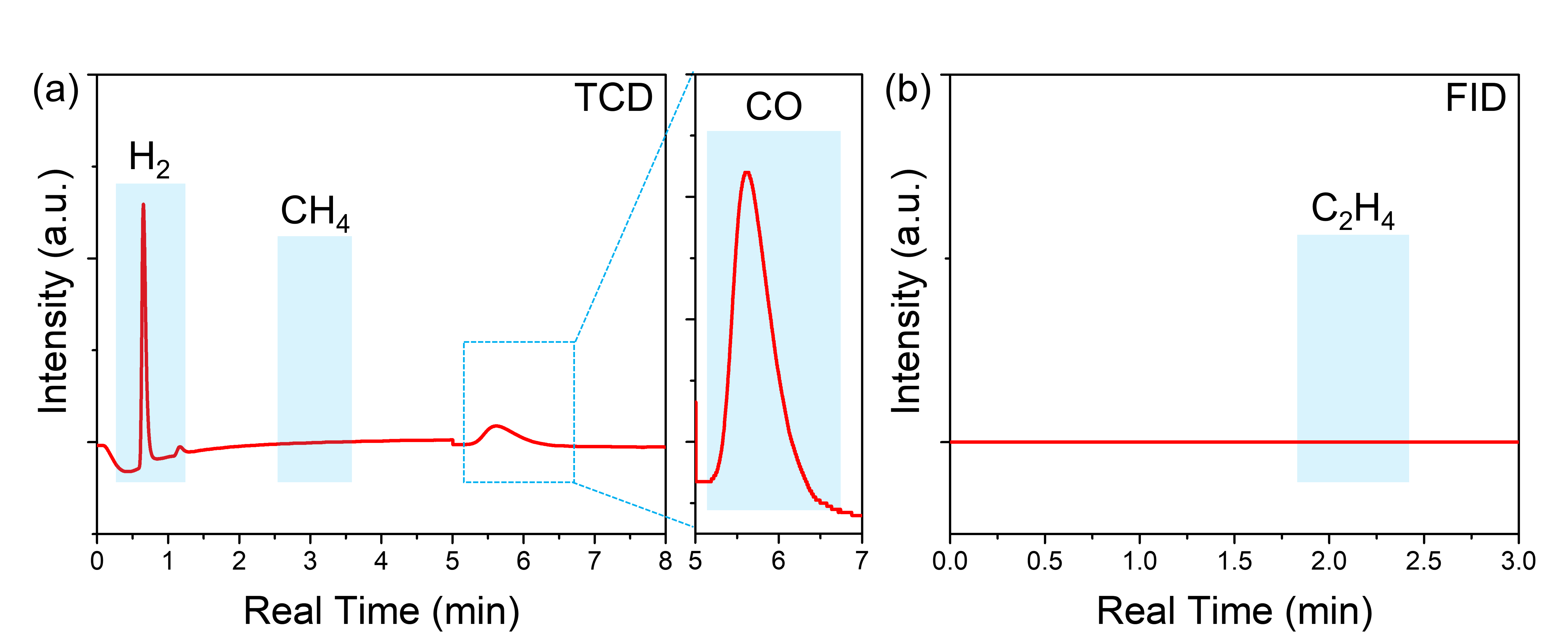

**Figure S5.** Typical TCD (a) and FID (b) patterns were measured by GC-FID for the products obtained using the CoPc/CP electrode with a loading of ~0.2 mg/cm² at an applied potential of -0.94 V vs. RHE for CO_2_ reduction in 1.0 M KOH. The peak area for CO is 2105, while C_2_H_4_ and CH_4_ are both 0. Using the method described in Section S-1.5, the Faradaic efficiency of CO was calculated to be approximately 99%, indicating that CO is the primary ECR product. More detailed peak areas and key values are provided in Table S3.

**Table S3.** The detailed key values for the products obtained using the CoPc/CP electrode with a loading of ~0.2 mg/cm² at various applied potentials for CO_2_ reduction in 1.0 M KOH.

| ***E* (V vs. RHE)** | ***J*_total_ (mA cm^-2^)** | **Peak area (CO)** | **Peak area (H_2_)** | **FE_H2_**  **(%)** | **FE_CO_**  **(%)** | ***J*_H2_ (mA cm^-2^)** | ***J*_CO_ (mA cm^-2^)** |
| --- | --- | --- | --- | --- | --- | --- | --- |
| -0.31 | -13.5 | 541 | 0 | 99.9 | 0 | -13.5 | 0.0 |
| -0.40 | -26.3 | 778 | 223 | 14.8 | 84.9 | -3.9 | -22.3 |
| -0.57 | -92.0 | 1736 | 513 | 1.4 | 98.3 | -1.3 | -90.4 |
| -0.70 | -165.1 | 6229 | 1276 | 2.1 | 97.6 | -3.5 | -161.1 |
| -0.83 | -235.7 | 1732 | 2043 | 0.4 | 99.5 | -0.9 | -234.5 |
| -0.94 | -332.8 | 2105 | 2799 | 0.4 | 99.3 | -1.3 | -330.5 |
| -1.04 | -394.8 | 5676 | 3449 | 0.7 | 99 | -2.8 | -390.9 |
| -1.13 | -493.5 | 66272 | 4040 | 3.1 | 96.9 | -15.3 | -478.2 |
| -1.21^#^ | -842.2 | 13315.6 | 1162.4 | 4.3 | 95.6 | -36.2 | -805.1 |
| -1.27^#^ | -1060.0 | 18856.5 | 1171.6 | 6.4 | 93.4 | -67.8 | -990.0 |
| -1.32^#^ | -1147.4 | 32398.4 | 1280.8 | 9.4 | 90.3 | -107.9 | -1036.1 |
| -1.38^#^ | -1266.3 | 83412.6 | 1103.7 | 24 | 76 | -303.9 | -962.4 |

**Note:** The total current density (*J*_total_) values were obtained from the current density-time curves shown in Figure S4. The peak areas from the GC-FID patterns, as well as the Faradaic efficiency (FE) and partial current density values, were calculated using the method described in Section S-1.5. The right shoulder marked with ^#^ used 30 sccm CO_2_ gas at 0.5 cm^2^ surface area, while the others used 15 sccm at 1.1 cm^2^ surface area.

**Table S4.** Comparation of the key-values in this work and literature, serving as a supplement to Table 1.

| **Catalyst** | **Electrolyte** | **FE_CO_**  **(%)** | ***E*_1_**  **(V)** | ***J*_CO_**  **(mA cm^-2^)** | ***E*_2_**  **(V)** | **Loading**  **(mg cm^-2^)** | **Ref.** |
| --- | --- | --- | --- | --- | --- | --- | --- |
| CoPc crystals | 1.0M KOH | >98.0 | -0.56 ~  -1.04 | -1036 | -1.32 | 0.2 | This work* |
| CoPc/CNT | 0.5M KHCO_3_ | 90.0 | -1.0 | -50 | -1.10 | - | ^[12]^ |
| CoPc/CNT | 0.1M KHCO_3_ | 98.0 | -0.63 | -15 | -0.63 | 0.4 | ^[13]^ |
| CoPc/CNT | H_2_SO_4_+K_2_SO_4_ | 73.0 | - | -38 | - | 0.8 | ^[14]^ |
| CoPc/CB | 1.0M KHCO_3_ | 98.0 | -1.05 | -100 | -0.65 | - | ^[15]^ |
| CoPc/CNT-MDE | 0.1M KHCO_3_ | 97.0 | - | -200 | - | - | ^[16]^ |
| CoPc/CNT-ODA | 0.5M KHCO_3_ | 97.7 | -1.0 | -350 | -1.3 | 1.0 | ^[17]^ |
| CoPc-TBG/CNT | 1.0M KOH | 96.0 | -0.72 | -112 | -0.72 | 0.2 | ^[18]^ |
| CoPc- EtO_8_/CNP | 1.0M KHCO_3_ | 95.0 | -2.20 | -340 | -2.20 | - | ^[19]^ |
| CoPc-OCH_3_/CNT | 0.5M KHCO_3_ | 97.0 | -1.0 | -280 | -1.10 | - | ^[12]^ |
| CoPc-NO_2_/CNT | 0.5M KHCO_3_ | 95.0 | -0.9 | -80 | -1.10 | - | ^[12]^ |
| CoPPc/CNT | 0.5M KHCO_3_ | 90.0 | -0.60 | -19 | -0.60 | 1.0 | ^[20]^ |
| NiPc/CNT-MDE | 0.5M KHCO_3_ | >98.0 | -0.54 ~ -0.68 | -400 | -0.70 | 0.4 | ^[21]^ |
| NiPc/NHCSs | 0.5M KHCO_3_ | 98.6 | -0.87 | -25 | -1.05 | 1.0 | ^[22]^ |
| NiPc(OH)_6_(DCNFO)/CNT | 1.0M KOH | >98.0 | -0.80 ~ -1.40 | -380 | -1.40 | - | ^[23]^ |
| NiPc-OMe | H_2_SO_4_+K_2_SO_4_ | 98.0 | -1.15 | -400 | -1.45 | 1.0 | ^[24]^ |

**Note: FE_CO_:** the maximum faradic efficiency for CO; ***E*_1_**: the potentials (vs RHE) of maximum FE_CO_; ***J*_CO_:** the maximum current density for CO; ***E*_2_**: the potentials (vs RHE) of maximum *J*_CO_; **CNT:** carbon nanotube; **CB:** carbon black; **MDE:** molecularly-dispersed electrocatalyst; **ODA:** octadecylamine; **TBG:** three tert-butyl groups; **CoPPc:** cobalt poly-phthalocyanine; **NHCS:** nitrogen-doped hollow carbon nanospheres.


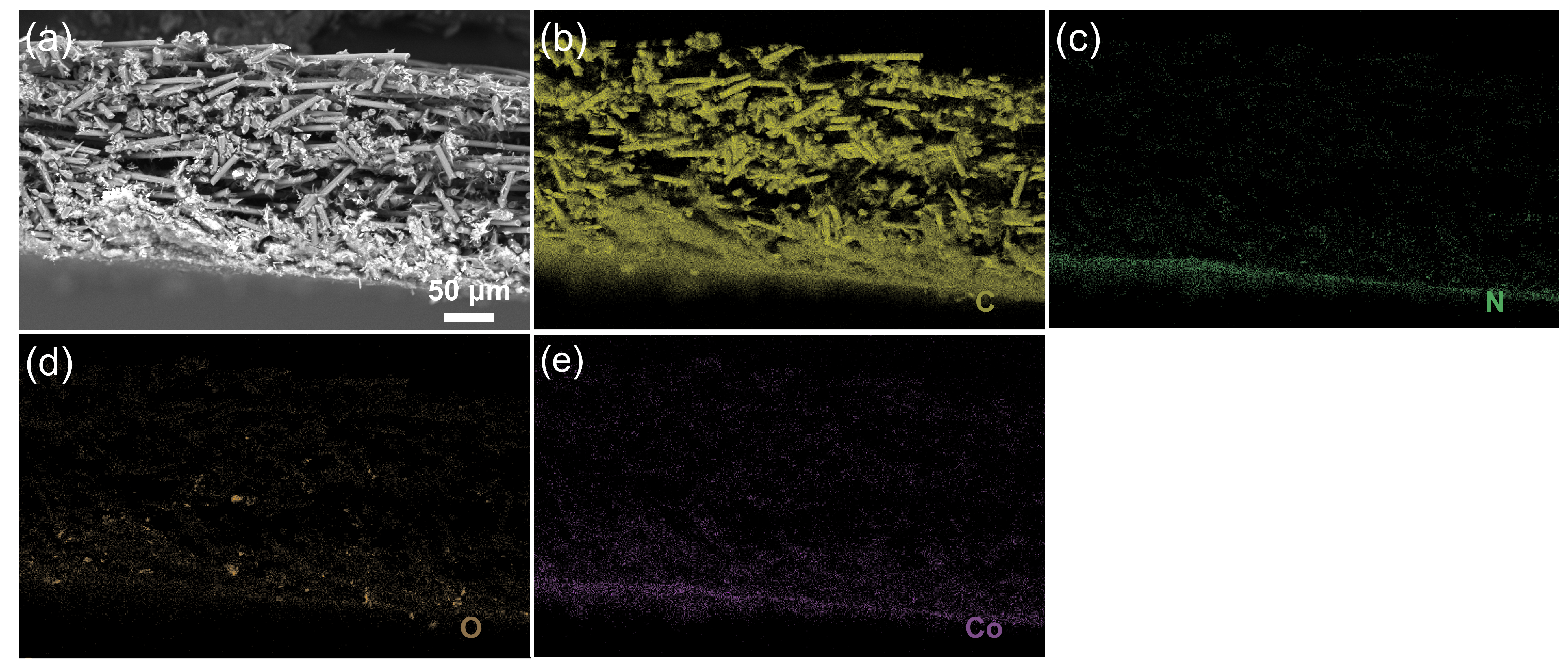


**Figure S6.** Cross-sectional SEM and elemental mapping images of CoPc crystals on the CP electrode before CO_2_RR. (a) SEM image, (b) Carbon mapping, (c) Nitrogen mapping, (d) Oxygen mapping, and (e) Cobalt mapping.


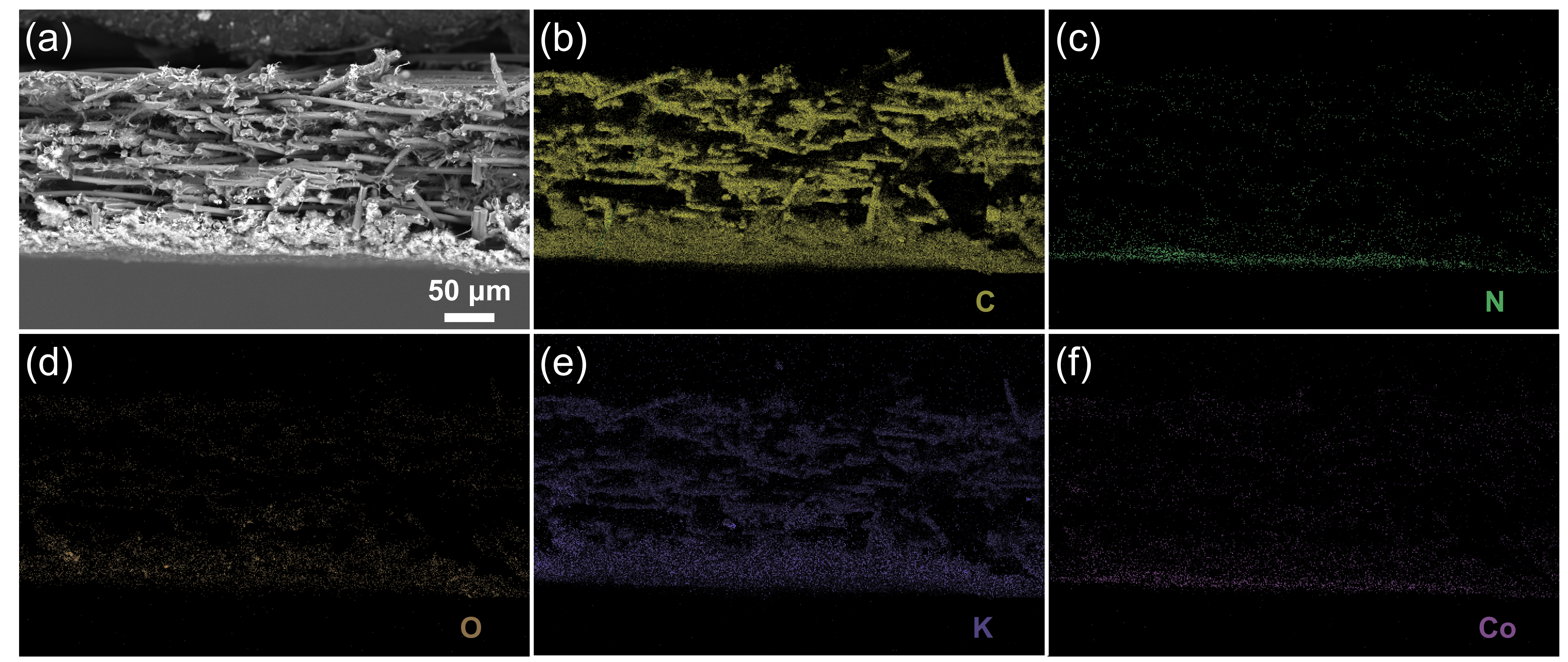


**Figure S7.** Cross-sectional SEM and elemental mapping images of CoPc crystals on the CP electrode after CO_2_RR. (a) SEM image, (b) Carbon mapping, (c) Nitrogen mapping, (d) Oxygen mapping, (e) Potassium mapping, and (f) Cobalt mapping.


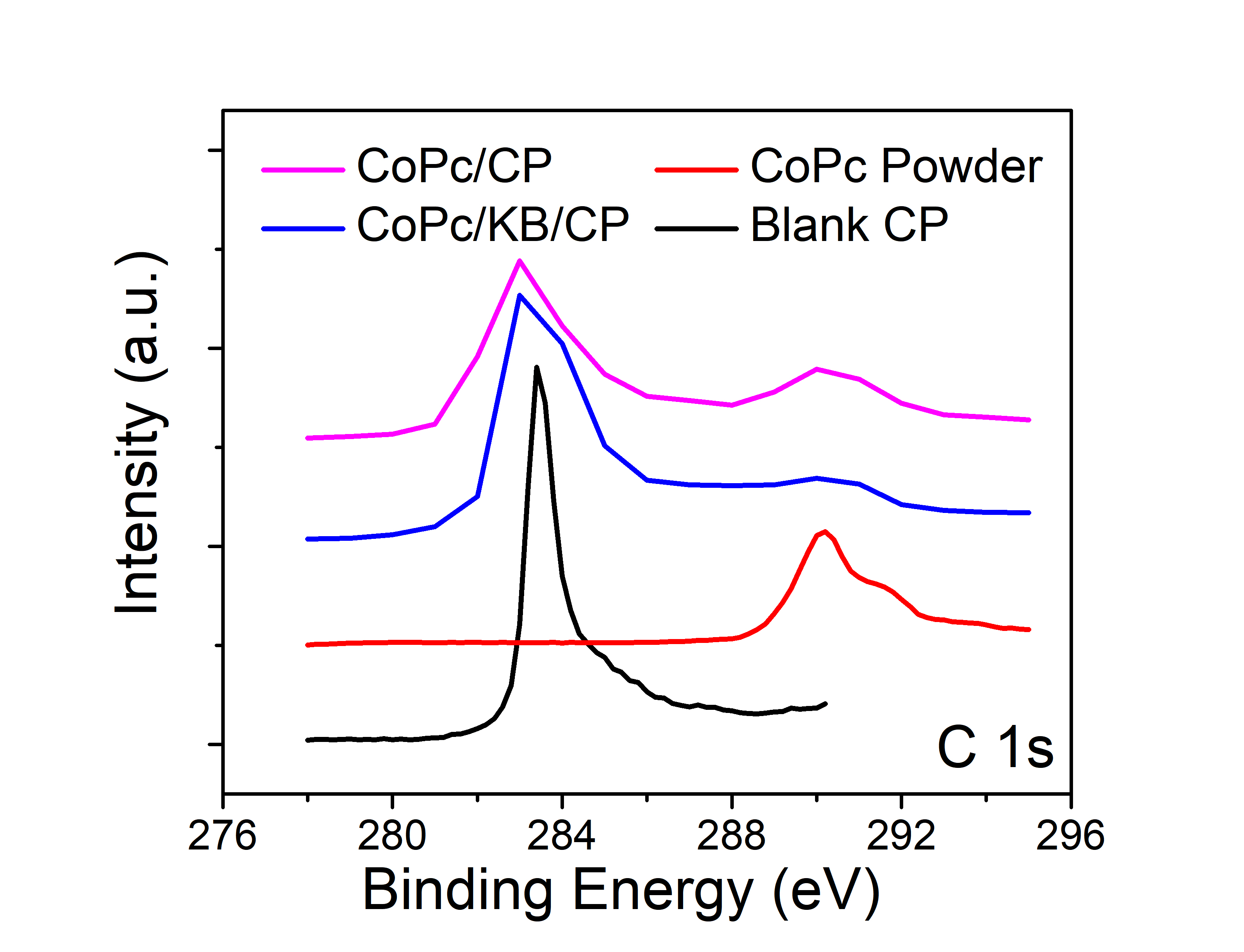


**Figure S8.** Typical XPS spectra of the C 1s region for CoPc/CP, CoPc/KB/CP electrodes, CoPc powder, and blank carbon paper. The peak at a binding energy of approximately 284 eV corresponds to carbon species from the carbon substrate, while the peaks at 288 to 293 eV corresponds to carbon species from the organic macrocyclic structure of phthalocyanine.


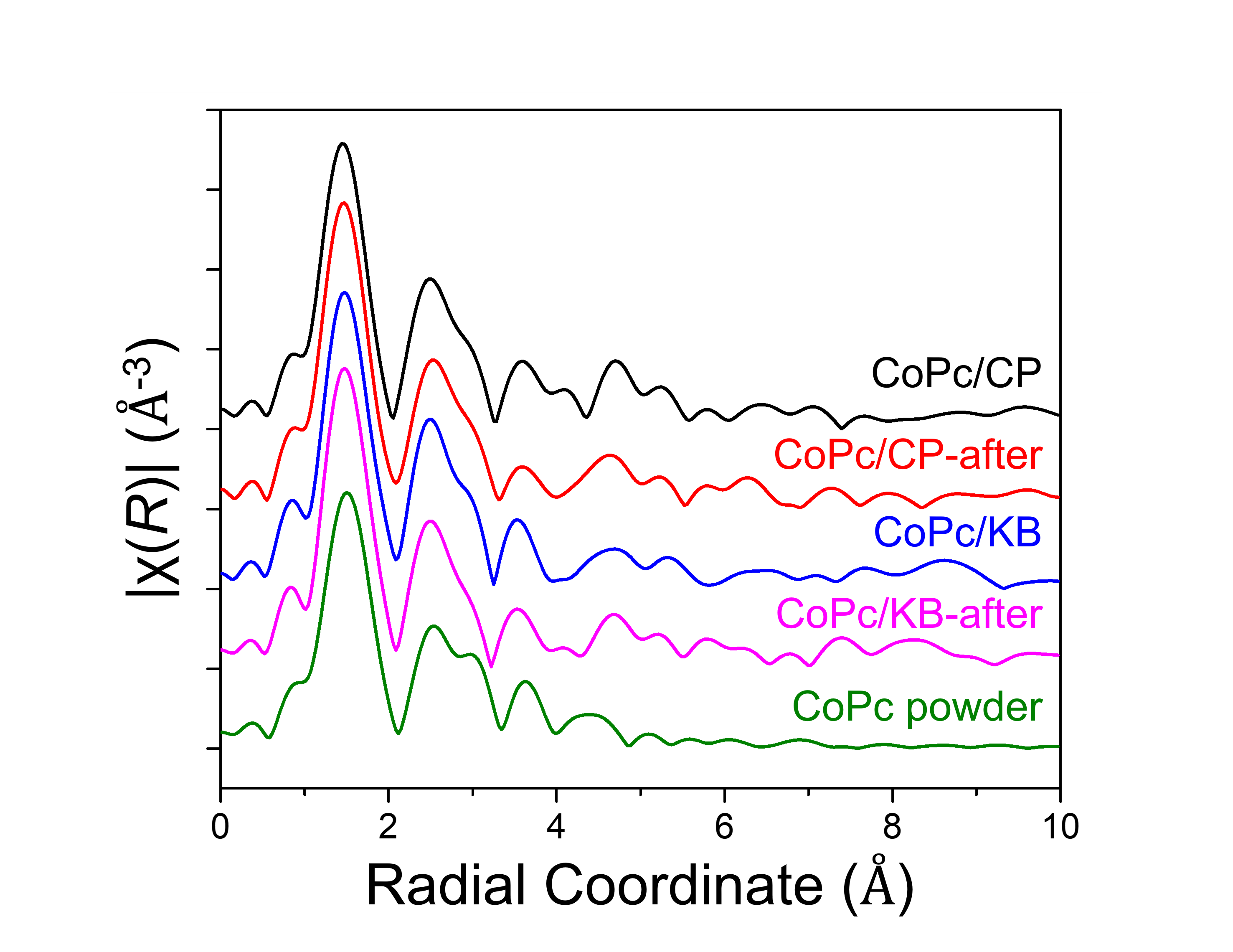


**Figure S9.** EXAFS spectra of CoPc/CP, CoPc/KB/CP electrodes before and after CO_2_RR, and CoPc powder.


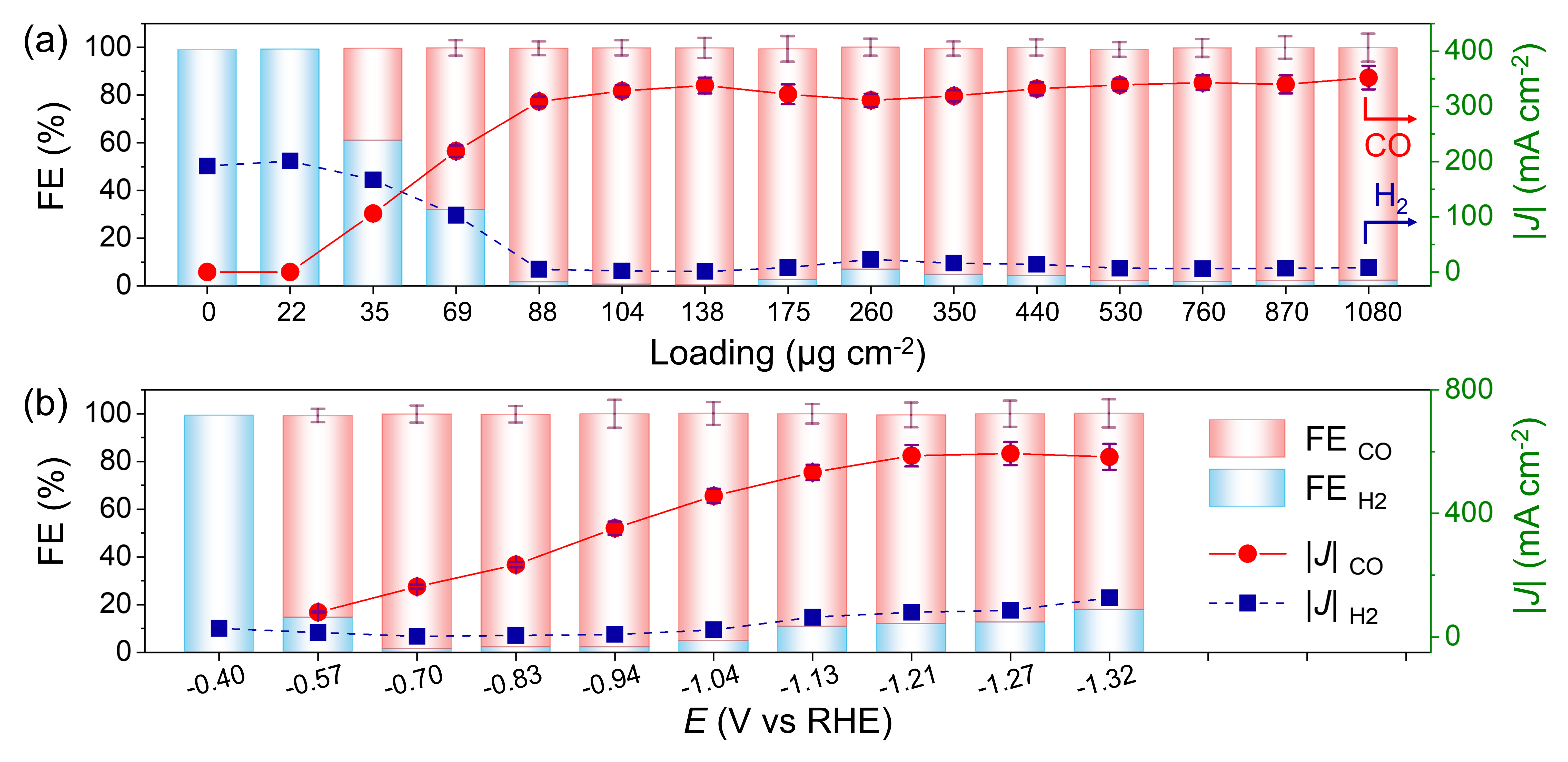
**Figure S10.** Electrochemical performance of various CoPc/KB GDEs for ECR. (a) Faradic efficiency (FE, left axis) and absolute current density (|*J*|, right axis) for CO (red) and H_2_ (blue) for electrodes at different loadings of CoPc/KB, at a potential (*E*) of -0.94V (vs RHE). (b) FE (left axis) and |*J*| (right axis) as a function of *E* (vs. RHE) for electrodes at a loading of ~1000 μg cm^-2^.


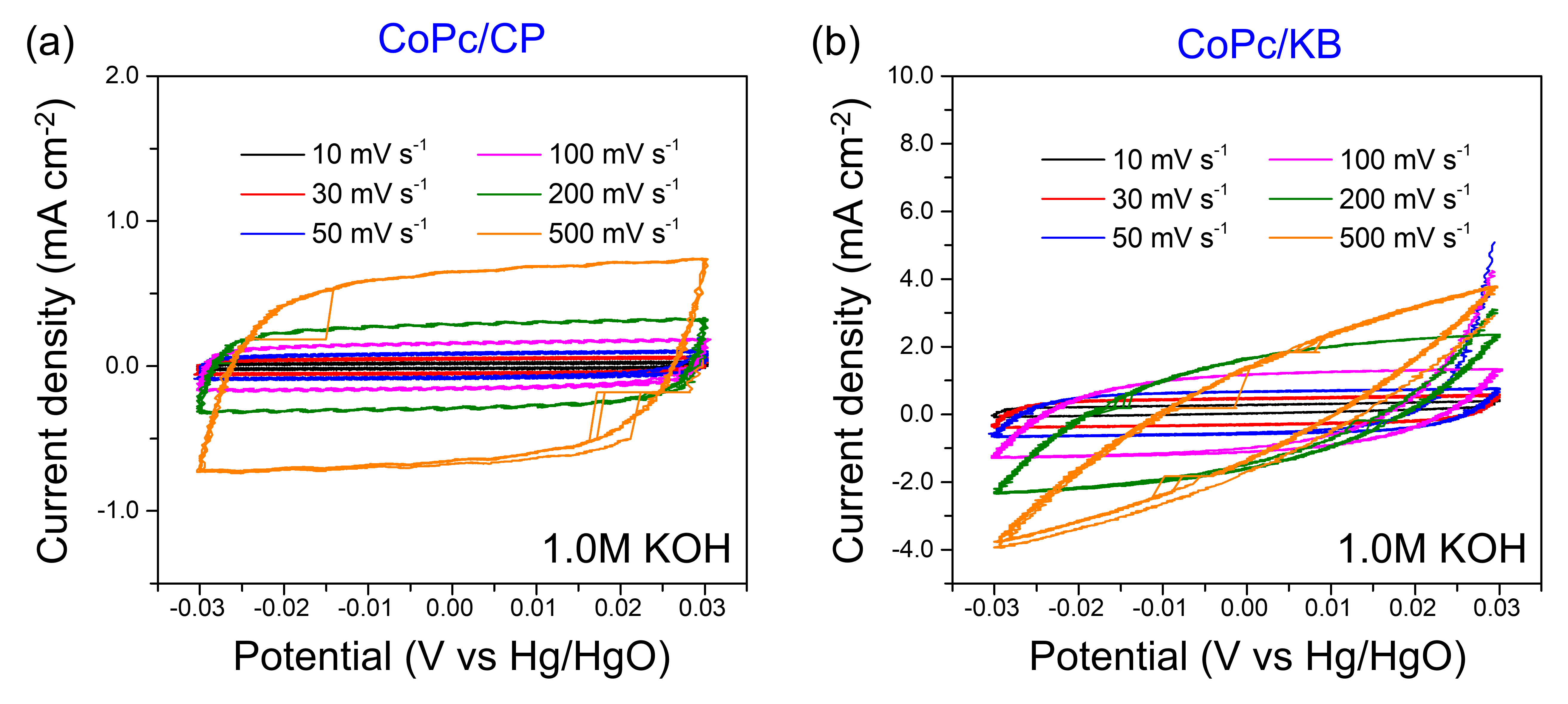
**Figure S11.** The cyclic voltammetry (CV) curves using (a) CoPc/CP and (b) CoPc/KB electrodes in 1M KOH solution at various scan rates, ranging from -0.03 to 0.03 V vs. Hg/HgO.


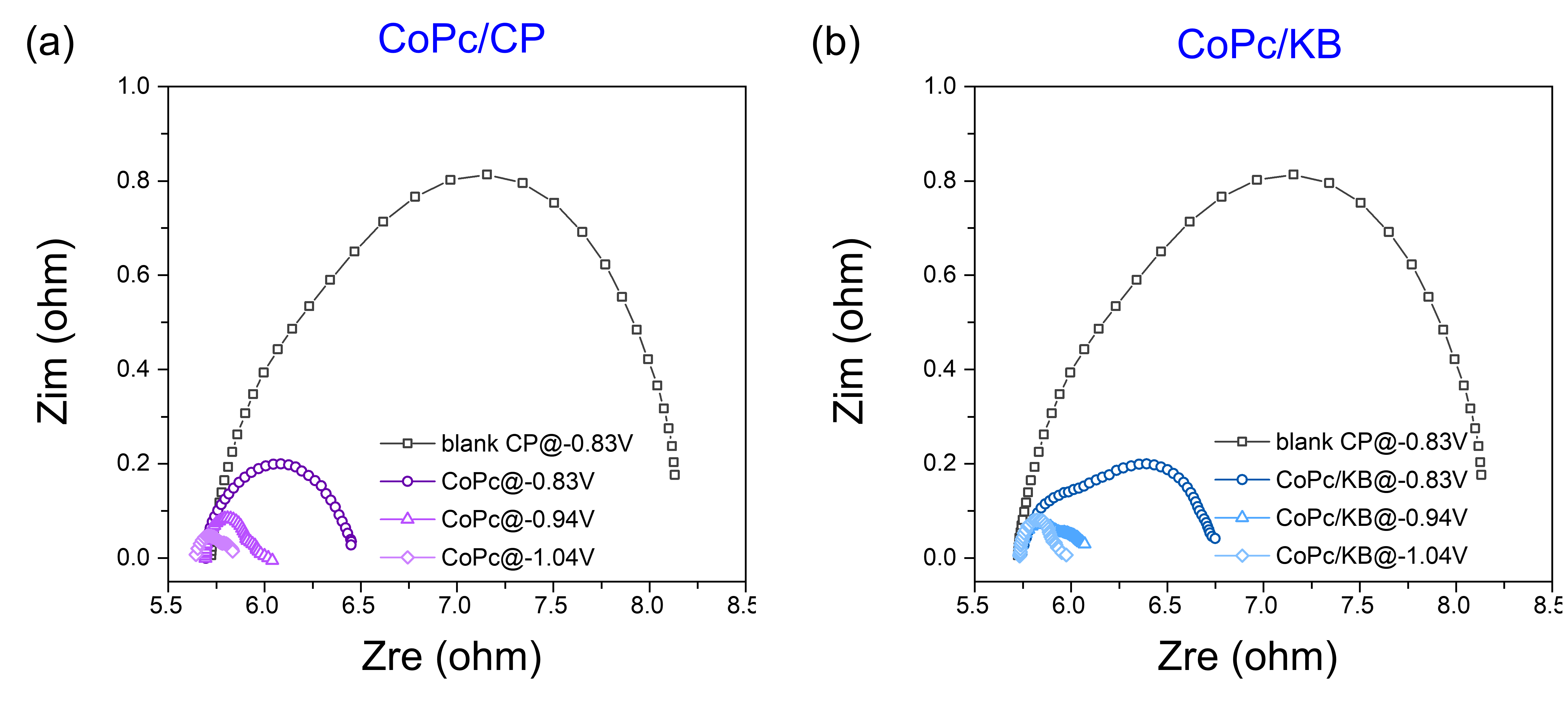
**Figure S12.** The *in situ* electrochemical impedance spectroscopy (EIS) measurements for (a) CoPc/CP and (b) CoPc/KB electrodes in 1M KOH solution at various potentials.


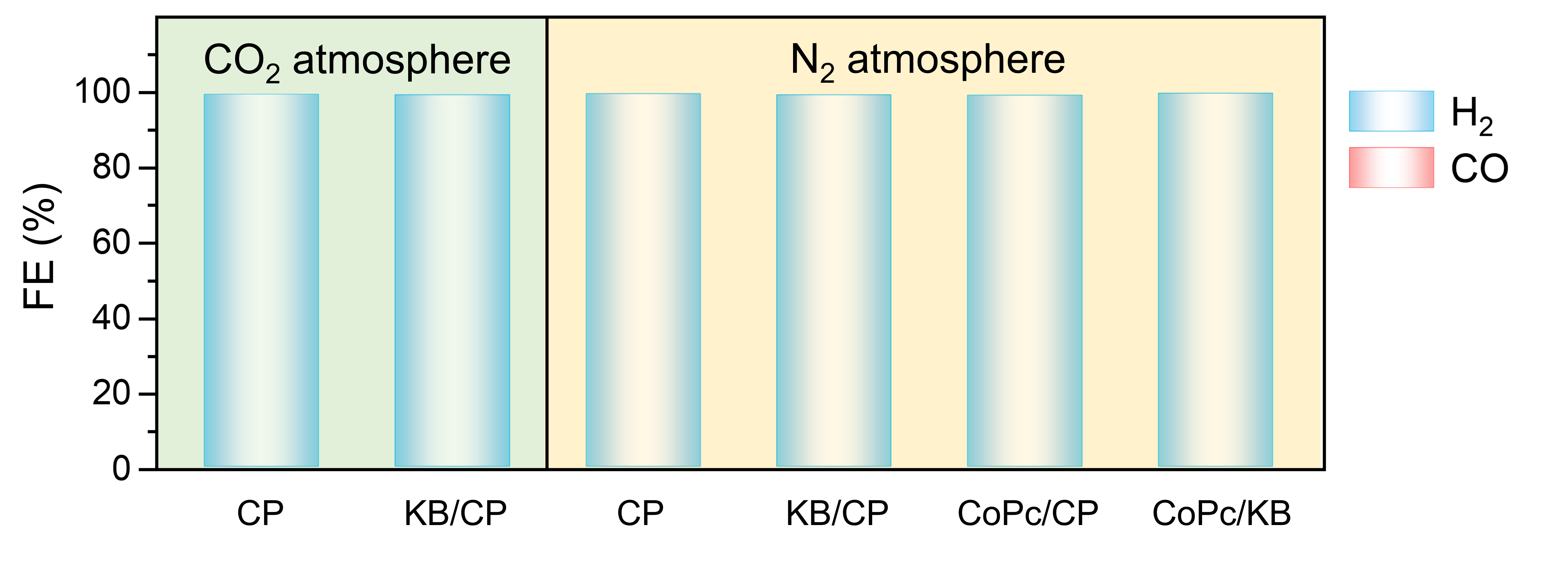
**Figure S13.** The electrochemical performance of (a) blank CP and KB/CP under a CO_2_ atmosphere and (b) blank CP, KB/CP, CoPc/CP, and CoPc/KB electrodes under an N₂ atmosphere in 1 M KOH solution at a current density of -100 mA cm^-2^. **Note:** Blank CP and KB/CP electrodes without catalyst produced only H_2_ under both CO_2_ and N_2_ atmospheres, indicating that KB exhibits no CO_2_RR activity. Similarly, CoPc and CoPc/KB tested under an N_2_ atmosphere yielded only H_2_, confirming that the products originated from the hydrogen evolution reaction (HER). These findings conclusively rule out the possibility of CO originating from KB or carbon paper.


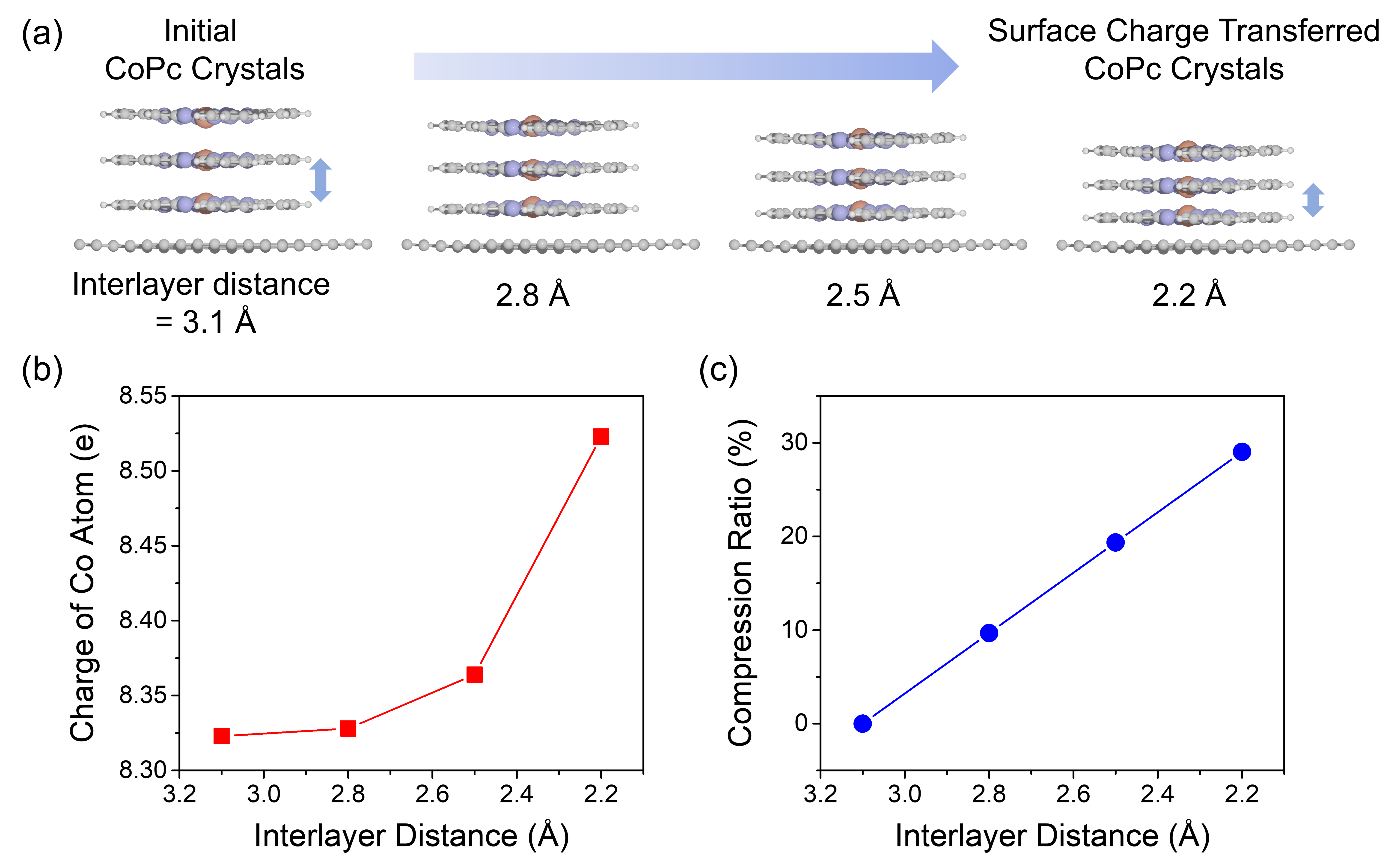


**Figure S14.** Qualitative simulation of surface charge transfer (SCT) in CoPc crystals by compressing the interlayer distance. (a) The initial CoPc structure has an interlayer distance of 3.1 Å, which is reduced to 2.8 Å, 2.5 Å, and 2.2 Å to simulate charge changes at Co sites. (b) The charge on Co atoms obviously increases with decreasing interlayer distance. (c) The compression ratio is calculated as the difference between the initial distance (3.1 Å) and the compressed distance (e.g., 2.2 Å), divided by the initial distance, yielding a compression ratio of about 29%. **Note:** In M-Pc molecules, the central metal atom acts as the sole electrocatalytic site, with intermediates adsorbing directly onto it. Our DFT calculations model adsorption energy perpendicular to the molecular plane, independent of crystal facets. Thus, the model captures CoPc's electrocatalytic behavior without considering facet effects.

**Supplementary Reference：**

[1] H. Abe, Y. Hirai, S. Ikeda, Y. Matsuo, H. Matsuyama, J. Nakamura, T. Matsue, H. Yabu, *NPG Asia Mater.* **2019**, *11*, 57.

[2] T. Liu, K. Ohashi, K. Nagita, T. Harada, S. Nakanishi, K. Kamiya, *Small* **2022**, *18*, 2205323.

[3] K. Kawashima, R. A. Márquez, Y. J. Son, C. Guo, R. R. Vaidyula, L. A. Smith, C. E. Chukwuneke, C. B. Mullins, *ACS Catal.* **2023**, *13*, 1893.

[4] Z. Yue, C. Ou, N. Ding, L. Tao, J. Zhao, J. Chen, *ChemCatChem* **2020**, *12*, 6103.

[5] T. Liu, H. Yabu, *EcoEnergy* **2024**, *2*, 419.

[6] B. Hammer, L. B. Hansen, J. K. Nørskov, *Phys. Rev. B - Condens. Matter Mater. Phys.* **1999**, *59*, 7413.

[7] J. Y. Jung, J. H. Park, Y. J. Jeong, K. H. Yang, N. K. Choi, S. H. Kim, W. J. Kim, *Korean J. Physiol. Pharmacol.* **2006**, *10*, 289.

[8] K. Li, L. Luo, Y. Zhang, W. Li, Y. Hou, *ACS Appl. Mater. Interfaces* **2018**, *10*, 41525.

[9] A. Allouche, *J. Comput. Chem.* **2012**, *32*, 174.

[10] K. Chan, C. Tsai, H. A. Hansen, J. K. Nørskov, *ChemCatChem* **2014**, *6*, 1899.

[11] W. Yang, Z. Jia, B. Zhou, L. Chen, X. Ding, L. Jiao, H. Zheng, Z. Gao, Q. Wang, H. Li, *ACS Catal.* **2023**, *13*, 9695.

[12] M. Huang, B. Chen, H. Zhang, Y. Jin, Q. Zhi, T. Yang, K. Wang, J. Jiang, *Small Methods* **2024**, *2301652*, 45.

[13] X. Zhang, Z. Wu, X. Zhang, L. Li, Y. Li, H. Xu, X. Li, X. Yu, Z. Zhang, Y. Liang, H. Wang, *Nat. Commun.* **2017**, *8*, 14675.

[14] S. Feng, X. Wang, D. Cheng, Y. Luo, M. Shen, J. Wang, W. Zhao, S. Fang, H. Zheng, L. Ji, X. Zhang, W. Xu, Y. Liang, P. Sautet, J. Zhu, *Angew. Chemie* **2024**, *63*, e202317942.

[15] M. Wang, A. Loiudice, V. Okatenko, I. D. Sharp, R. Buonsanti, *Chem. Sci.* **2023**, *14*, 1097.

[16] X. Wu, J. W. Sun, P. F. Liu, J. Y. Zhao, Y. Liu, L. Guo, S. Dai, H. G. Yang, H. Zhao, *Adv. Funct. Mater.* **2022**, *32*, 2107301.

[17] L. Xiong, X. Fu, Y. Zhou, P. Nian, Z. Wang, Q. Yue, *ACS Catal.* **2023**, *13*, 6652.

[18] M. Wang, K. Torbensen, D. Salvatore, S. Ren, D. Joulié, F. Dumoulin, D. Mendoza, B. Lassalle-Kaiser, U. Işci, C. P. Berlinguette, M. Robert, *Nat. Commun.* **2019**, *10*, 3602.

[19] S. Ren, E. W. Lees, C. Hunt, A. Jewlal, Y. Kim, Z. Zhang, B. A. W. Mowbray, A. G. Fink, L. Melo, E. R. Grant, C. P. Berlinguette, *J. Am. Chem. Soc.* **2023**, *145*, 4414.

[20] N. Han, Y. Wang, L. Ma, J. Wen, J. Li, H. Zheng, K. Nie, X. Wang, F. Zhao, Y. Li, J. Fan, J. Zhong, T. Wu, D. J. Miller, J. Lu, S. T. Lee, Y. Li, *Chem* **2017**, *3*, 652.

[21] X. Zhang, Y. Wang, M. Gu, M. Wang, Z. Zhang, W. Pan, Z. Jiang, H. Zheng, M. Lucero, H. Wang, G. E. Sterbinsky, Q. Ma, Y. G. Wang, Z. Feng, J. Li, H. Dai, Y. Liang, *Nat. Energy* **2020**, *5*, 684.

[22] S. Gong, W. Wang, R. Lu, M. Zhu, H. Wang, Y. Zhang, J. Xie, C. Wu, J. Liu, M. Li, S. Shao, G. Zhu, X. Lv, *Appl. Catal. B Environ.* **2022**, *318*, 121813.

[23] Y. Jin, X. Zhan, Y. Zheng, H. Wang, X. Liu, B. Yu, X. Ding, T. Zheng, K. Wang, D. Qi, J. Jiang, *Appl. Catal. B Environ.* **2023**, *327*, 122446.

[24] Z. Jiang, Z. Zhang, H. Li, Y. Tang, Y. Yuan, J. Zao, H. Zheng, Y. Liang, *Adv. Energy Mater.* **2023**, *13*, 2203603.
